# Microcirculation Dysfunction in Subacute Stroke: The Role of Delayed Capillary Pericyte Loss

**DOI:** 10.14336/AD.2025.0197

**Published:** 2026-06-04

**Authors:** Yiya Xu, Chao Chen, Jilin Weng, Ting Chen, Yingchao He, Zhiwei Song, Yinzhou Wang

**Affiliations:** ^1^Shengli Clinical Medical College of Fujian Medical University, Fuzhou, Fujian, China.; ^2^Department of Neurology, Fuzhou University Affiliated Provincial Hospital, Fuzhou, Fujian, China.; ^3^Fujian Key Laboratory of Medical Analysis, Fujian Academy of Medical Sciences, Fuzhou, Fujian, China.; ^4^Department of Neurology, Wuyishan Municipal Hospital, Wuyishan, Fujian, China.

**Keywords:** fasudil, ischemic stroke, microcirculation, necroptosis, pericyte

## Abstract

Post-recanalization microcirculation dysfunction is common and significantly contributes to poor outcomes in ischemic stroke. Pericytes have been shown to mediate the “no-reflow” phenomenon by constricting capillaries in experimental stroke models, implicating their critical role in early microcirculation dysfunction. However, little is known about the long-term fate of pericytes and their contribution to sustained microcirculation dysfunction in prolonged period of time. We conducted repeated longitudinal observations of pericyte fate and function, as well as blood flow dynamics across multiple vascular segments, using two-photon imaging in PDGFRβ-tdTomato mice subjected to transient middle cerebral artery occlusion (tMCAO) over a 14-day period. Multivariate analysis was performed to identify imaging features independently associated with capillary perfusion on day 14. Types of pericyte death were assessed using immunohistochemistry and Western blot analysis. Fasudil and the RIPK1 inhibitor necrostatin-1 were administered to modulate pericyte dysfunction and survival during the acute and subacute phases of stroke. Outcomes were evaluated by total capillary perfusion, infarct volume, blood brain barrier (BBB) integrity, and neurological function over 14 days. Pericyte loss observed on day 7 post-stroke was independently associated with impaired microcirculation perfusion, as indicated by a reduction in total capillary volume. While fasudil treatment alone improved microcirculation perfusion on day 3, it did not alter pericyte fate or improve outcomes by day 14. Necroptosis was found to contribute to delayed pericyte loss in the ischemic penumbra. Combined therapy with fasudil and necrostatin-1 effectively prevented delayed pericytes loss and improved both microcirculation perfusion and neurological outcomes on day 14. Delayed pericyte loss contributes to irreversible microcirculation dysfunction in the subacute phase of stroke. Targeting pericyte dysfunction and necroptosis following recanalization represents a promising therapeutic strategy for enhance stroke recovery.

## INTRODUCTION

Ischemic stroke (IS) is one of the leading causes of death and disability worldwide [1, 2]. Currently, early recanalization of occluded arteries through intravenous thrombolysis or interventional thrombectomy is the most promising strategy for treating acute IS. However, in at least 50% of patients, timely recanalization of proximal arteries fails to restore perfusion in the downstream microcirculation. This results in severe complications, such as reperfusion injury, worsening intracerebral edema, or hemorrhagic transformation [3, 4]. The mechanisms underlying futile recanalization are not fully understood. Several mechanisms have been implicated, including capillary constriction, endothelial swelling, microthrombosis, and the formation of neutrophil extracellular traps (NETs) [5-7]. Increasing evidence suggests that capillary pericyte dysfunction plays a key role in mediating the impaired microcirculation following recanalization [8-10].

Capillary pericytes are vascular mural cells with distinctive surface markers and morphology, located intermittently along the outer walls of capillaries. Physiologically, pericytes play crucial roles in maintaining the structural integrity of the blood-brain barrier and regulating regional blood perfusion through their ability to constrict and dilate [8, 11]. Under pathological conditions such as stroke, pericytes constrict the capillaries in response to regional ischemia, leading to microcirculation dysfunction [8, 9]. Notably, pericyte-mediated constriction can persist even after recanalization of upstream vessels [9, 10, 12, 13]. Previous studies using fasudil, a Rho-kinase inhibitor with calcium antagonistic properties, have shown that preventing pericyte constriction results in immediate capillary dilation and improved capillary perfusion in ischemic brain, indicating a potential strategy for treating impaired microcirculation [6, 8, 10, 14]. However, these studies have primarily focused on pericyte dysfunction within the first 24 hours after ischemia-reperfusion [6, 14], corresponding to the acute phase of stroke. The fate of capillary pericytes during prolonged period of time after stroke, particularly in the subacute phase, remains unclear. Furthermore, the mechanisms underlying pericyte loss following sustained constriction are not fully understood. Exploring the role and fate of pericytes throughout the entire recovery phase of ischemic stroke is of great significance for developing new therapeutic strategies targeting pericyte dysfunction.

We hypothesize that capillary pericytes undergo significant dysfunction and loss during both the acute and subacute phases of stroke, which correlates with impaired microcirculation in the perifocal region. Moreover, interventions aimed at preserving pericyte function and promoting their survival may improve post-recanalization microcirculation and neurological outcomes in experimental stroke.

In this study, we performed repeated *in vivo* imaging to monitor dynamic changes in pericyte function and survival in fluorescently labeled mice to investigate the role of pericyte dysfunction in stroke outcomes. Additionally, we examined the mechanisms underlying pericyte loss following ischemia-reperfusion and evaluated the efficacy of therapeutic interventions targeting pericyte dysfunction and survival.

## MATERIALS AND METHODS

### Animal experiments

All animal experiments were conducted in accordance with the Guide for the Care and Use of Laboratory Animals published by the National Institutes of Health (NIH) and were approved by the Animal Ethics Committee of the Clinical Trial Center, Fujian Provincial Hospital. The study adhered to the ARRIVE guidelines to ensure rigorous and reproducible reporting. Male PDGFRβ-Cre/ERT and Rosa-LSL-tdTomato mice, aged 8–12 weeks, were purchased from the Shanghai Model Organisms Center. Only male animals were used to minimize sex-related variability. Mice were randomly assigned to experimental groups and housed in a specific pathogen-free (SPF) environment with a 12-hour light/dark cycle and free access to food and water. Tamoxifen (75 mg/kg) was administered intraperitoneally for five consecutive days to induce tdTomato expression in pericytes via Cre recombinase activation. For all surgical procedures, mice were anesthetized with isoflurane to minimize discomfort (4–5% for induction and 2–3% for maintenance). In this study, all the outcome assessments related to intervention and data analysis were performed in a blinded manner.

### Laser speckle contrast imaging determination

Cerebral blood flow (CBF) was measured using laser speckle contrast imaging (LSCI; RFLSI Pro, Reward) at baseline and at multiple time points up to 14 days post-reperfusion. CBF values were normalized to baseline and expressed as percentages. The ischemic core was defined as the regions with CBF < 30% of baseline, and the perifocal region as the area with CBF between 30% and 50% of baseline.

### Cranial window for blood flow measurement

Cranial window surgery was performed under isoflurane anesthesia, with mice secured in a stereotaxic frame for stability. We first used LSCI to evaluate regional CBF and localize the penumbra zone in a separate cohort of mice (n=3) without cranial window. The penumbra zone was defined as brain regions with a CBF reduction to 30-50% of baseline after tMCAO, as previously reported (Supplementary Fig. 1). Based on the LSCI result, a 4-mm-diameter cranial window was created over the penumbra region (centered at -2 mm bregma, 1.5 mm lateral, left hemisphere) using a high-speed drill. A sterile glass coverslip was placed over the exposed brain tissue and sealed with tissue adhesive (Vetbond) to maintain optical clarity. A custom-designed titanium head ring was affixed to the skull using dental cement to enable stable imaging during two-photon microscopy. The body temperature was maintained at 37°C throughout the procedure. Mice were given a two-week recovery period to allow for adequate healing before imaging. During recovery, animals were monitored daily for signs of distress or complications.

### A mouse model of transient middle cerebral artery occlusion

Transient middle cerebral artery occlusion (tMCAO) was performed in 8–12-week-old male mice to induce focal cerebral ischemia. Following a midline neck incision under isoflurane anesthesia, the left common carotid artery and external carotid artery were exposed. A silicone-coated nylon filament (diameter: 0.18 ± 0.01 mm) was gently advanced through the internal carotid artery to occlude the middle cerebral artery (MCA). Occlusion was maintained for 60 minutes, after which the filament was carefully withdrawn to allow reperfusion. The body temperature was maintained at 37°C throughout the procedure using a heating pad. Mice were monitored postoperatively, and those with severe complications were excluded from further analysis.

### *In vivo* two-photon imaging assessment

Mice were injected intravenously with 0.1 mL of 10 mg/mL FITC-dextran (2,000,000 Da, Sigma-Aldrich, St. Louis, MO) to label vasculature. After 15 minutes, light anesthesia (0.25–0.75% isoflurane) was maintained, and body temperature was kept at 37°C. Mice were immobilized on a custom-built stage. Two-photon imaging during the ischemic phase was initiated within 15 minutes after MCA occlusion and required 30-40 minutes to complete the session. A two-photon microscopy was performed using a ×25 water immersion objective (Olympus FVMPE) and a tunable pulsed Chameleon infrared two-photon laser (Coherent) with an excitation wavelength of 920 nm. Emission signals were collected using BA495-540 (green) and BA575-645 (red) band-pass filters. Z-stack images of pericytes were acquired at 1-µm intervals. Their number, morphology, associated vascular diameters, and perfused capillary volume were analyzed using Imaris software (version 9.0.1). Hemodynamic parameters, such as red blood cell (RBC) velocity, were measured using line scans repeated 1,000 times per vessel. Post-acquisition XYZ drift correction was applied to all images using Huygens software (version 23.10.0).

For each mouse, three regions of interest (ROIs; 509µm*509µm) were selected within the cranial window based on perfusion of cortical branches of the MCA as assessed by LSCI and were followed throughout the study. At each imaging time point, the same fields were localized based on the pattern of surface pial arteries and veins, along with the overall vascular architecture (Supplementary Fig. 2).

### Tissue collection and immunohistochemistry

Mice were euthanized under deep isoflurane anesthesia, followed by transcardial perfusion with cold PBS and fixation in 4% paraformaldehyde (PFA). Brains were post-fixed in 4% PFA overnight, cryoprotected in 30% sucrose, embedded in OCT compound, sectioned at a thickness of 20µm, and stored at -20°C. Immunohistochemistry was performed using primary and secondary antibodies, as detailed in Supplementary Table 1. Appropriate negative controls were included to validate antibody specificity.

### Western blotting analysis

Brain tissues were homogenized in RIPA lysis buffer containing protease and phosphatase inhibitors. Proteins were separated by SDS-PAGE, transferred to PVDF membranes, and probed with primary antibodies against p-MLKL, p-RIP1, MLKL, and RIP1. Band intensities were quantified relative to total protein expression.

### 2,3,5-Triphenyltetrazolium chloride (TTC) staining

To assess infarct volume, brains were sectioned into 2-mm-thick coronal slices, stained with 2% TTC at 37°C for 15–30 minutes, and subsequently fixed in 4% PFA. Infarct volume was quantified using ImageJ software.

### Evans blue extravasation

Evans blue extravasation was used to evaluate blood-brain barrier (BBB) integrity. Following intravenous injection of 2% Evans blue, brain tissues were homogenized, and dye concentration was measured spectrophotometrically at 610 nm. Results were expressed as Evans blue content (µg) per gram of tissue.

### IgG leakage determination

IgG staining was used to evaluate extravascular IgG leakage in brain tissue sections. Sections were stained with an anti-mouse IgG antibody and an avidin biotin enzyme complex reagent (Vector Labs, Burlingame, CA). Diaminobenzidine (DAB) was used for signal visualization. For each section, four representative fields from the peri-infarct region were imaged. The average Integrated Optical Density (IOD) of IgG staining was calculated using Image J software.

### Neurological function evaluation

Neurological function was assessed using the modified Neurological Severity Score (mNSS), which evaluates motor, sensory, balance, and reflex functions. Assessments were performed during ischemia, and on day 7 and 14 post-reperfusion by blinded observers.

### Pharmacological intervention

Fasudil hydrochloride (MedChem Express, USA) was administered intraperitoneally at a dose of 10 mg/kg once daily for 14 days post-reperfusion. Necrostatin (Nec-1; Targetmol, USA) was administered intraperitoneally at a dose of 1.65mg/kg once daily for 14 days, starting at the time of reperfusion.

### Statistical analysis

Statistical analyses were performed using GraphPad Prism (version 9.3.1). Data are presented as mean±SD, and normality was assessed using the Shapiro-Wilk test. Statistical methods included linear mixed-effects models, one-way ANOVA or two-way ANOVA, Mann-Whitney U test, and LASSO regression. A *p*-value < 0.05 was considered statistically significant.

**Figure 1.**
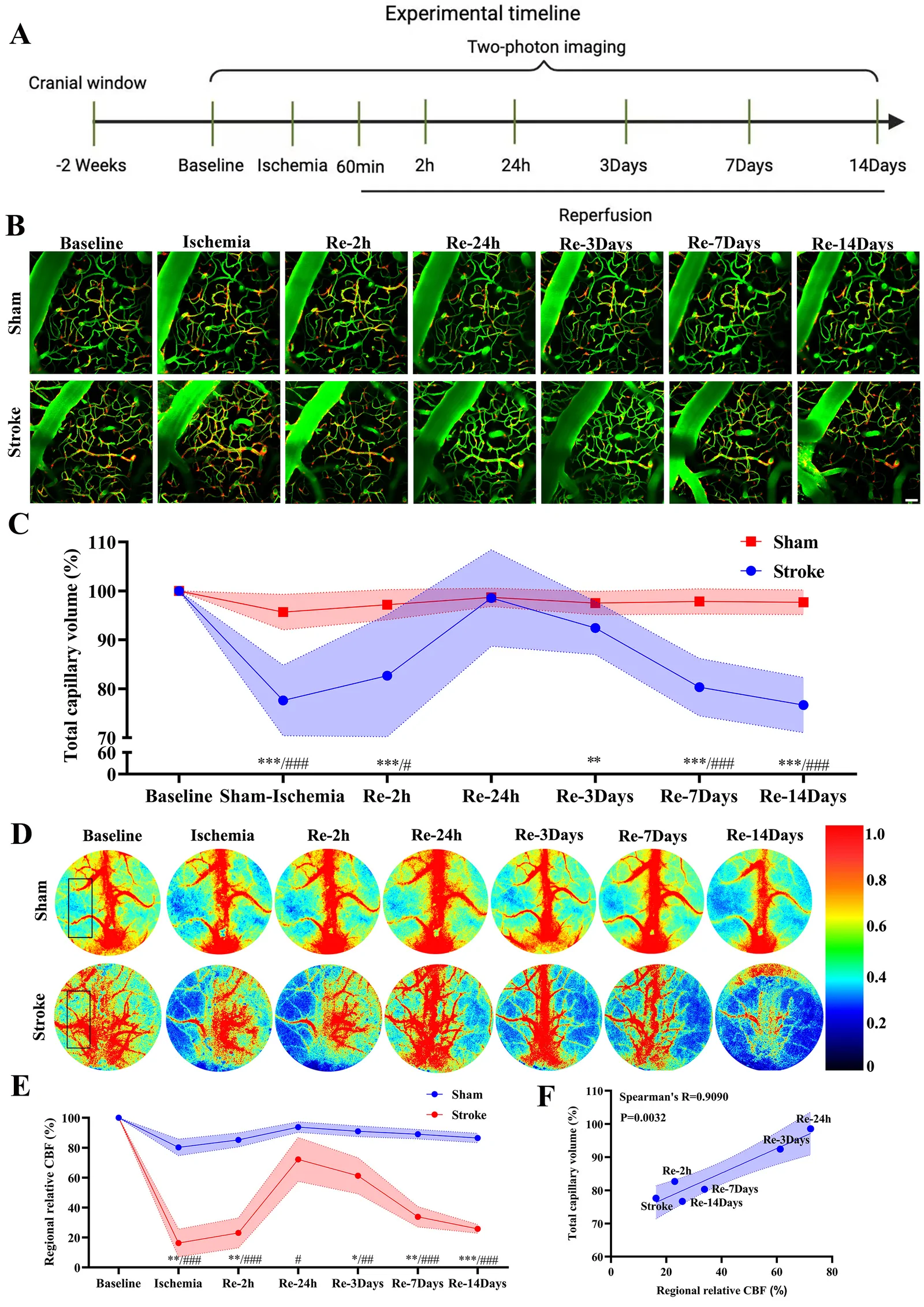
**Assessment of capillary perfusion and its correlation with cerebral blood flow (CBF) following tMCAO. (A)** Schematic representation of the experimental timeline for in vivo two-photon imaging. Panel created with Biorender.com. **(B)** Representative image showing changes in total capillary volume (TCV) in the ischemic penumbra zone captured by two-photon imaging. Scale bar = 50 µm. **(C)** Quantification and comparison of TCV changes between sham and stroke groups. *A linear mixed-effects model was used to compare each post-stroke time point (Ischemia, Re-2h, Re-24h, Re-3Days, Re-7Days and Re-14Dyas) to baseline in the stroke group. #Two-way ANOVA was used to compare TCV between stroke and sham groups at each time point. N = 16 for the stroke group; N = 4 for the sham group. **(D)** Representative laser speckle contrast image following tMCAO. The black box indicates the region of interest (ROI) used for CBF quantification. **(E)** Quantification and comparison of regional relative CBF changes in the sham and stroke groups. *One-way ANOVA was used to compare each post-stroke time point to baseline in the stroke group. #Two-way ANOVA was used to compare regional relative CBF between the sham and stroke groups at each time point. N = 4 for the stroke group; N = 4 for the sham group. **(F)** Spearman’s rank correlation between TCV and regional relative CBF, based on mean values at 7 time points. * or # *p* < 0.05; ** or ## *p* < 0.01; *** or ### *p* < 0.001. Data are presented as mean ± SD. Abbreviations: Baseline, before ischemia; Re-2h, 2 hours after reperfusion; Re-24h, 24 hours after reperfusion; Re-3days, 3 days after reperfusion; Re-7 days, 7 days after reperfusion; Re-14days, 14 days after reperfusion; CBF, cerebral blood flow.

## RESULTS

### Baseline characterization of capillary pericytes and vascular hierarchy

Brain capillary pericytes were first visualized in PDGFRβ-tdTomato mice subjected to sham operations (n=4) using two-photon imaging. Cerebral vasculature was labeled with green fluorescence, while pericytes were marked with red fluorescence. Abundant PDGFRβ^+^ mural cells with diverse morphologies were observed along the outer walls of most vessels. These mural cells included both pericytes and smooth muscle cells (Supplementary Fig. 4A).

The cerebral vasculature was classified into seven segments based on vessel diameter, flow direction, and the morphology of the ensheathing mural cells. Pial and penetrating arteries, with average diameters of 24.19 ± 2.50 µm and 13.33 ± 2.22 µm respectively, were ensheathed by PDGFRβ^+^ mural cells with indistinct somas and circumferential processes encircling the lumen-features consistent with smooth muscle cells as reported in previous studies (Supplementary Figs. 4B, 4C). Pre-capillary arterioles, identified as the first branches of penetrating arteries, had an average diameter of 4.98 ± 0.51 µm and were ensheathed by mural cells with ovoid somas and ring-like or elongated processes (Supplementary Fig. 4D). The classification and role of mural cells in pre-capillary arterioles remain debated, as this population may include both smooth muscle cells and pericytes.

Capillaries branching from pre-capillary arterioles, with diameters averaging 4.08 ± 0.99 µm, constituted the majority of the microvascular network. These vessels were fully covered by mural cells with prominent ovoid somas and thin, strand-like processes oriented longitudinally along the lumen (Supplementary Fig. 4E), consistent with established definitions of pericytes. Post-capillary venules, identified based on diameter and flow direction, were sporadically covered by mural cells with shorter processes than those on pre-capillary arterioles. Ascending venules, which followed post-capillary venules, were wrapped by mural cells with circumferential processes (Supplementary Fig. 4F). Pial venules were ensheathed by mesh-like mural cells, previously characterized as venous smooth muscle cells (Supplementary Fig. 4G). In this study, capillary pericytes were selected for further analysis based on their morphology and specific vascular location.

### Observation of capillary perfusion alterations in the perifocal region following tMCAO in mice

Mice with implanted cranial windows (n=16) underwent a two-week recovery period before receiving tMCAO and two-photon imaging. Baseline characteristics of vasculature and pericytes in the perifocal region were evaluated prior to tMCAO. Two-photon imaging was performed at multiple time points, including baseline, ischemia, and 2 hours, 24 hours, 3 days, 7 days, and 14 days after reperfusion, to track changes in labeled vessels and cells (Fig. 1A). This study represents the longest in vivo imaging observation of cerebral vasculature following experimental stroke to data. Four mice died during the observation period, while the remaining twelve completed the follow-up. The total volume of visualized capillaries in the perifocal region was quantified to assess microcirculation perfusion (Fig. 1B). Following tMCAO, total capillary volume (TCV) significantly decreased to 77.62% ± 7.19% of baseline levels. Although nearly full recovery was observed at 24 hours post-reperfusion, TCV progressively declined from day 3 to day 14. By day 14, TCV had decreased to 76.67% ± 5.65% of baseline. No dynamic changes in TCV were observed in sham-operated mice (Fig. 1C). To assess cerebral blood flow, LSCI was also performed repeatedly in a separate group of mice subjected to tMCAO (Fig. 1D). CBF showed a similar biphasic trend over the 14-day follow-up period (Fig. 1E). Spearman correlation analysis revealed a strong positive correlation between TCV and CBF in the perifocal region (Spearman’s R=0.9090, p=0.0032) (Fig. 1F). These findings indicate that microcirculation perfusion undergoes a biphasic change after stroke, which is closely associated with regional blood flow dynamics.

**Figure 2.**
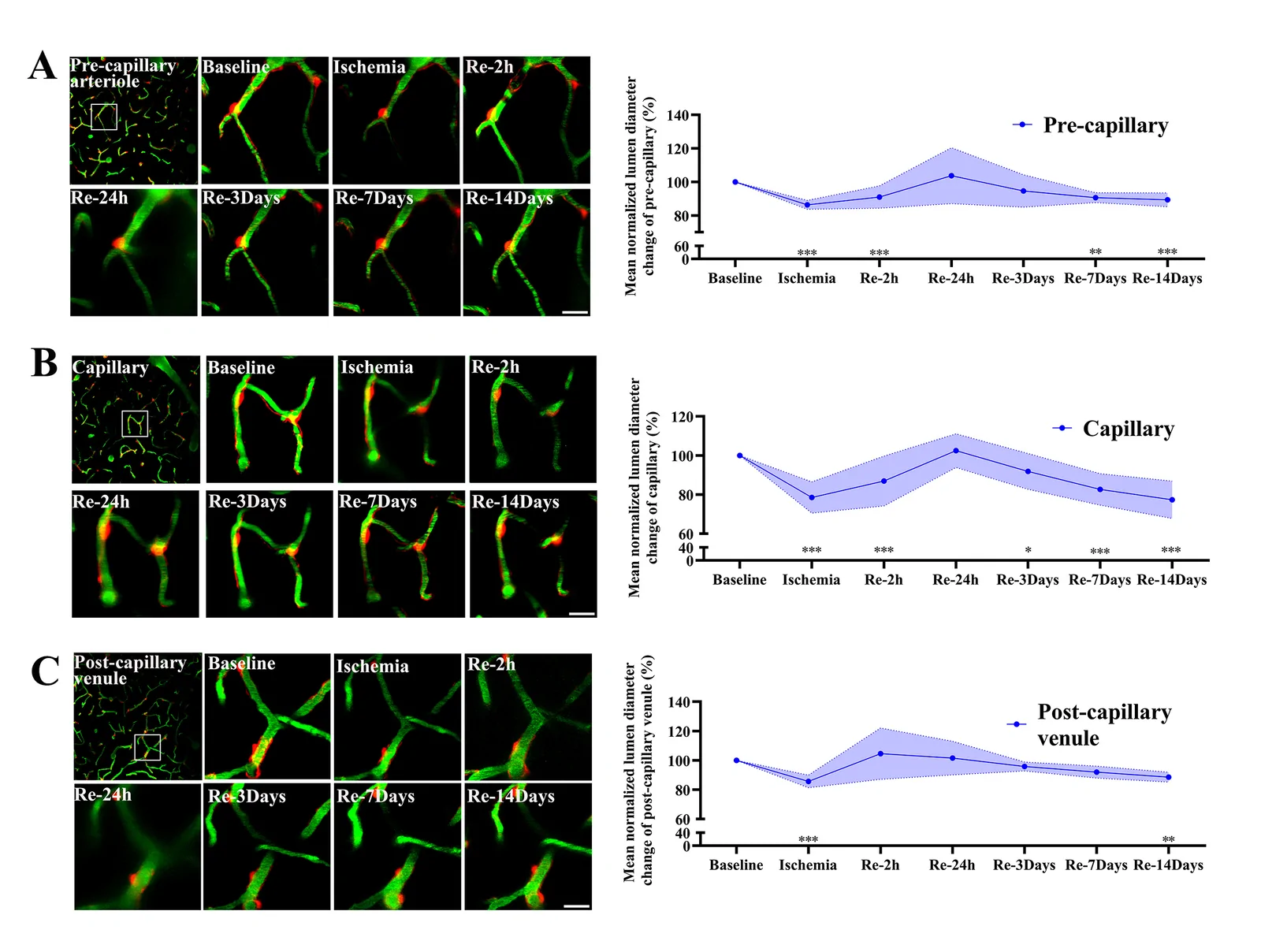
**Assessment of vascular segment diameter changes following tMCAO by two-photon imaging.(A-C)** Left panels: Representative two-photon images showing changes in different vascular segments, including pre-capillary arteriole, capillary, and post-capillary venule within the region of interest at seven time points following tMCAO. Scale bar = 20μm. Right panels: Quantification and comparison of mean normalized lumen diameter changes across different vascular segments. * A linear mixed-effects model was used to compare each post-stroke time point to baseline within the stroke group. N = 16 for the stroke group. *p < 0.05, ** *p* < 0.01, *** *p* < 0.001. Data are presented as mean ± SD.

### Capillary constriction associated with micro-circulation dysfunction

Previous studies have demonstrated that excessive pericyte constriction can impair microcirculation perfusion despite proximal artery reperfusion, leading to the “no-reflow” phenomenon. However, the specific vascular segments involved in the development of “no-reflow,” as well as the long-term consequences of microcirculation dysfunction, remain unclear. In this study, we observed changes in lumen diameter across different vessel segments at multiple time points following tMCAO and analyzed their correlation with capillary perfusion.

To determine whether these vascular changes were specific to ischemic injury, a sham-operated group was included for comparison. In the sham group, vessel lumen diameters remained stable over time in all vascular segments (Supplementary Fig. 5).

In the tMCAO group, the mean lumen diameters of most vessel segments, except for the pial artery, were significantly reduced during MCA occlusion. However, the patterns of diameter changes over the 14-day follow-up period varied by segments. Pre-capillary arterioles and capillaries showed similar trends: their lumen diameters decreased during ischemia and returned to baseline by 24 hours post-reperfusion. However, from day 3 to day 14, both segments exhibited progressive narrowing, with significant reductions observed on day 14 (Figs. 2A, B). Post-capillary venules also showed progressive narrowing during the subacute phase, with significant diameter reductions observed over time. (Figs. 2C). These findings suggest sequential impairment within consecutive microvascular segments, contributing to sustained perfusion deficits. Meanwhile, pial and penetrating arteries exhibited marked dilation after reperfusion, peaking at 24 hours post-tMCAO and gradually returning to baseline by day 14 (Supplementary Fig. 6A, B). Similarly, ascending venules and pial venules exhibited significant diameter reductions on day 14 (Supplementary Fig. 6C, D). The early-phase dilation of pial and penetrating arteries may reflect compensatory collateral circulation. However, the progressive constriction of capillaries, pre-capillary arterioles, and off-stream venous vessels suggests a persistent impairment of microcirculation function despite such compensation. Pearson correlation analysis revealed a strong correlation between changes in capillary lumen diameter and TCV after tMCAO (R^2^=0.9804, p=0.0006) (Supplementary Fig. 7). These findings indicate that excessive capillary constriction directly contributes to microcirculation dysfunction in experimental stroke.

**Figure 3.**
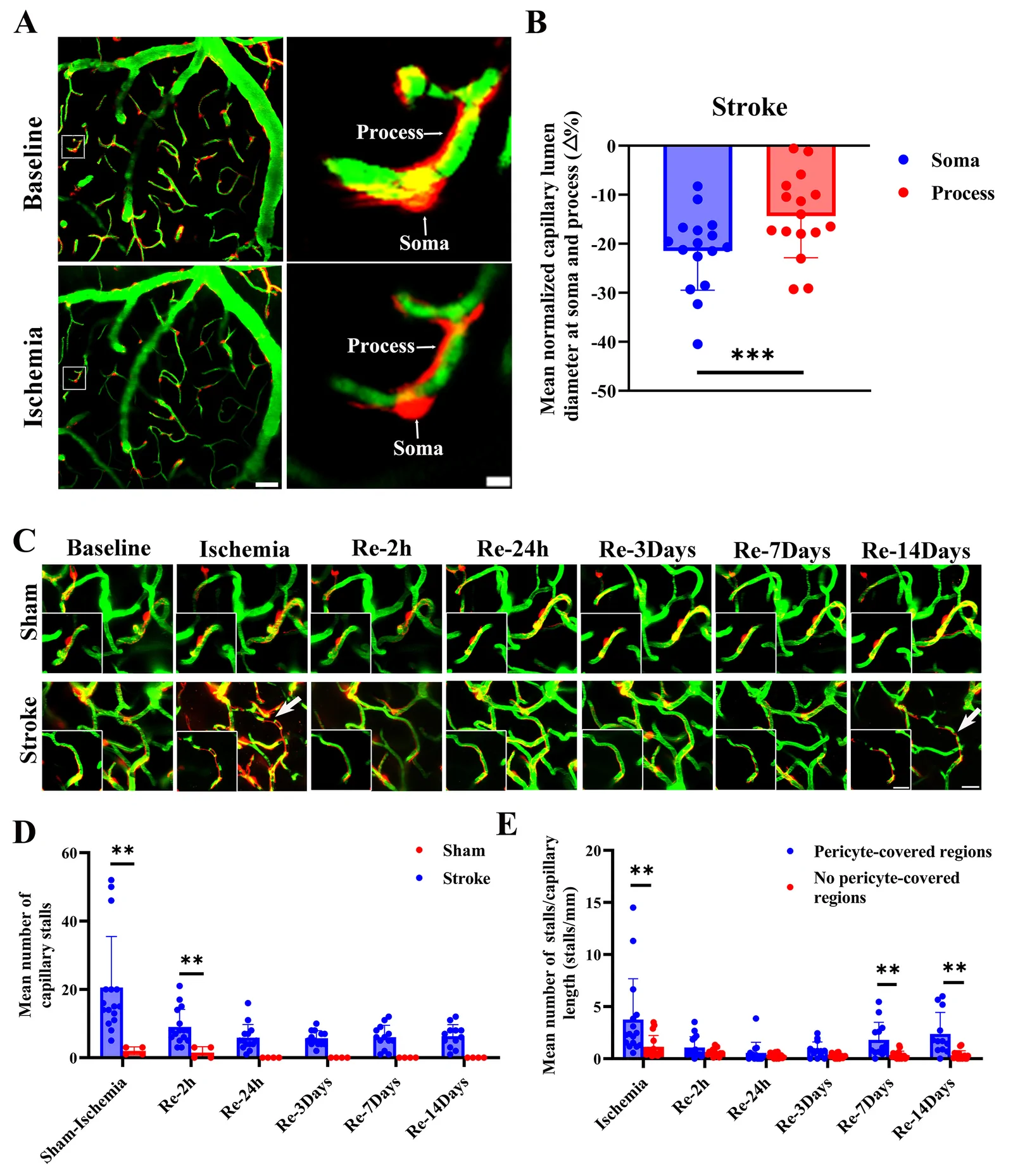
**Pericyte involvement in capillary stalling following tMCAO. (A)** Left: Z-projections of two-photon images showing capillary beds at baseline and during ischemia. Scale bar = 50 μm. Right: High-magnification images of individual capillary pericytes; white arrows indicate pericyte somas and processes. Scale bar = 5 µm. **(B)** Quantification of mean normalized capillary lumen diameter changes at pericyte somas or processes during ischemia relative to baseline. A linear mixed-effects model was used. N = 16 for the stroke the group. **(C)** Representative image showing capillary stalling following tMCAO, with white arrows indicating sites of stalling. Scale bar = 20 μm. **(D)** Quantification and comparison of the mean number of capillary stalls in sham and stroke groups. A Mann-Whitney U test was used for group comparison. **(E)** Quantification and comparison of mean number of stalls in pericyte-covered vessel segments versus uncovered vessel segments. A Mann-Whitney U test was used. N = 16 for the stroke group; N = 4 for the sham group. * *p* < 0.05, ** *p* < 0.01, *** *p* < 0.001. Data are presented as mean ± SD.

### Pericyte-mediated excessive capillary constriction and blood flow stasis following tMCAO in mice

To investigate whether pericytes contribute to capillary constriction during experimental stroke, we compared changes in mean lumen diameter at capillary sites covered by pericyte somas versus those covered by pericyte processes (Fig. 3A). Our findings revealed significant lumen narrowing at both types of sites after tMCAO. However, the reduction was more pronounced at sites associated with somas than at those associated with processes (Fig. 3B). Given that capillaries lack intrinsic contractility, these observations suggest that capillary constriction following tMCAO is mediated by pericyte contraction.

We also evaluated capillary stalls, blood flow velocity, and volume over time following stroke, as these are direct indicators of microcirculation impairments. Using two-photon imaging, capillary stalls appeared as segmental dark regions within the vessel lumen, caused by discontinuities in FITC-labeled plasma (Fig. 3C). The number of capillary stalls was significantly higher in tMCAO mice than in sham-operated mice during the follow-up period (Fig. 3D). Moreover, in the tMCAO group, capillary stalls occurred more frequently in vessel segments associated with pericytes than in uncovered segments (Fig. 3E). A significant reduction in blood flow velocity and volume was observed in capillary segments covered by pericyte somas, based on RBC influx measurements (Supplementary Fig. 8). Collectively, these results suggest that capillary pericytes play a critical role in microcirculation dysfunction by constricting capillaries following tMCAO. The impact of pericytes persisted throughout both the acute and subacute phases of experimental stroke.

### Delayed pericyte loss was associated with worsened microcirculation outcomes following tMCAO in mice

**Figure 4.**
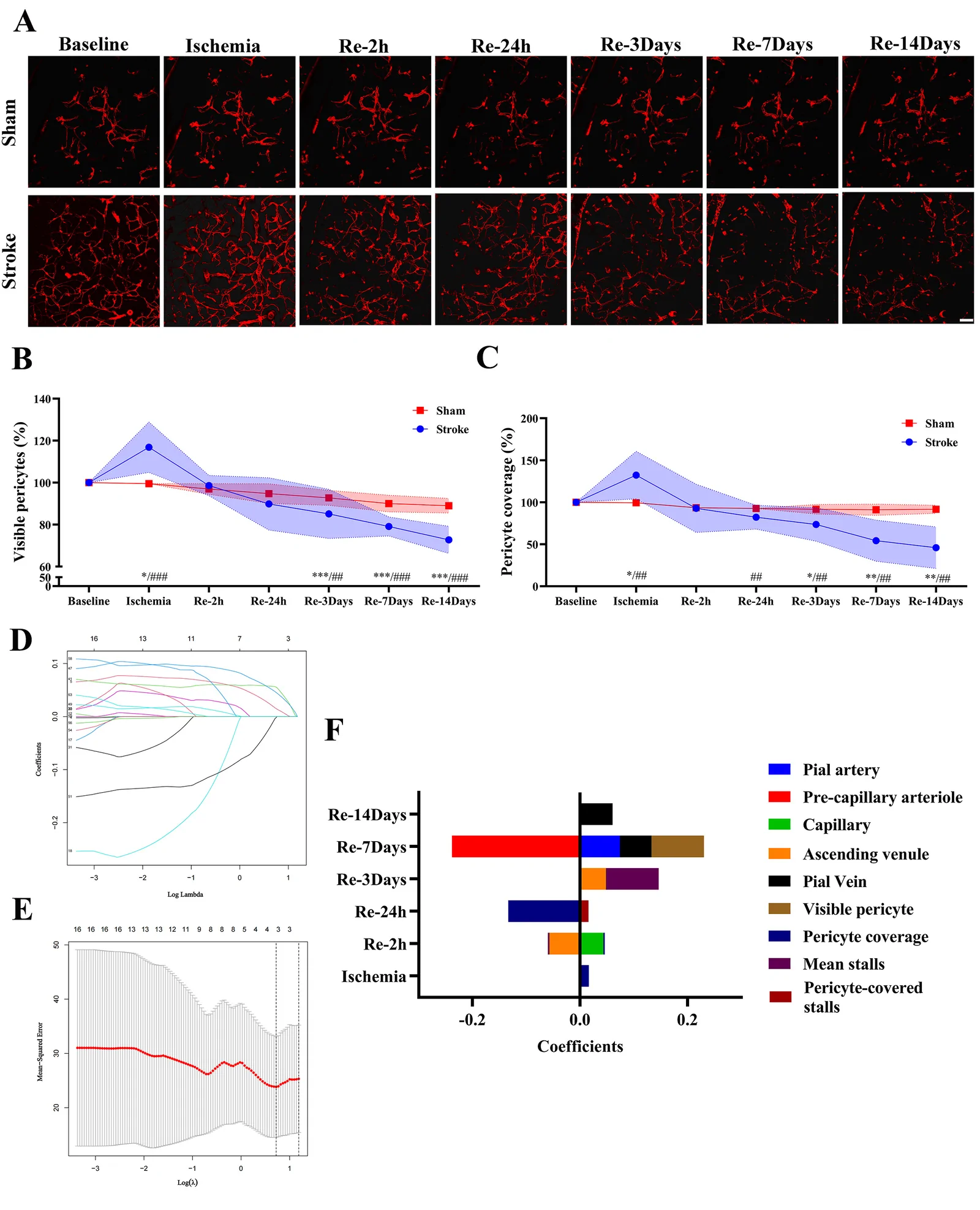
**Pericyte alterations and their association with microcirculation outcomes following tMCAO. (A)** Representative two-photon image showing changes in visible pericytes following tMCAO. Scale bar = 50μm. **(B)** Quantification and comparison of visible pericyte in sham and stroke groups. **(C)** Quantification and comparison of pericyte coverage in sham and stroke groups. *A linear mixed-effects model was used to compare each post-stroke time point to baseline within the stroke group. #Two-way ANOVA was used to compare visible pericyte and coverage between the sham and stroke groups at each time point. N = 16 for the stroke group; N = 4 for the sham group. **(D)** Selection of the optimal lambda (λ) value using 5-fold cross-validation. The minimum mean squared error (MSE) was observed at λ = 0.4119861. The Lasso coefficient profile for predictors was plotted against log(λ). **(E)** Cross-validation curve showing the MSE as a function of log (λ). Red dots represent the MSE at each λ value, vertical dashed lines indicate the λ with the minimum MSE and the λ determined by the one-standard-error rule. **(F)** Coefficient distribution of variables selected by Lasso regression. *or # *p* < 0.05, ** or ## *p* < 0.01, *** or ### *p* < 0.001. Data are presented as mean ± SD.

Previous studies have suggested that pericytes die in rigor, causing persistent capillary constriction after reperfusion. Here, we investigated changes in the number of visible pericytes over time following tMCAO and their association with microcirculation perfusion outcomes. Capillary pericytes were identified based on their morphology and the vessel segments they covered (Fig. 4A). We observed that the number of visible capillary pericytes increased shortly after stroke, returning to baseline levels two hours after reperfusion. Although a slight decrease in the number of visible pericytes was observed at 24 hours, the difference was not statistically significant. A progressive loss of visible pericytes was noted from day 3 to day 14. By day 14, the number of pericytes had declined to 71.41% ± 8.98% of the baseline level. In the sham-operated group, the number of visible pericytes remained stable over 14-day post-procedure period (Fig. 4B). Pericyte coverage of capillaries exhibited a similar trend during follow-up observations (Fig. 4C). Pericyte loss after experimental stroke has been reported in other studies using in vivo imaging. However, the transient increase in pericyte number observed in our study has not been previously reported. Considering that the time was too short for pericytes to replicate soon after ischemia, alternative mechanisms may be involved in the appearance of visible pericytes, such as perifocal activation or migration. Over time, excessive constriction and persistent regional hypoxia likely contributed to pericyte death. We performed a comprehensive analysis of the influence of multiple vessel-associated features on capillary perfusion on day 14 using LASSO regression. Lumen diameters of seven different vessel segments, changes in pericyte number and coverage, and the number of capillary stalls at different time points were included in the analysis (Figs. 4D, 4E). Our results showed that the number of visible pericytes on day 7 was positively associated with total capillary volume at day 14 (coefficient: 0.097). In contrast, pericyte coverage at 24 hours and increased lumen diameter of pre-capillary arterioles on day 7 were inversely associated with total capillary volume (coefficients: -0.133 and -0.238, respectively). These findings suggest that better pericyte survival during the subacute phase of stroke is protective for microcirculation perfusion. Conversely, increased pericyte coverage during the acute phase may contribute to worsened capillary perfusion due to excessive constriction. Taken together, this study highlights a biphasic regulatory role of pericyte function in microcirculation following stroke (Fig. 4F).

### Fasudil led to short-term improvements in microcirculation in experimental stroke

**Figure 5.**
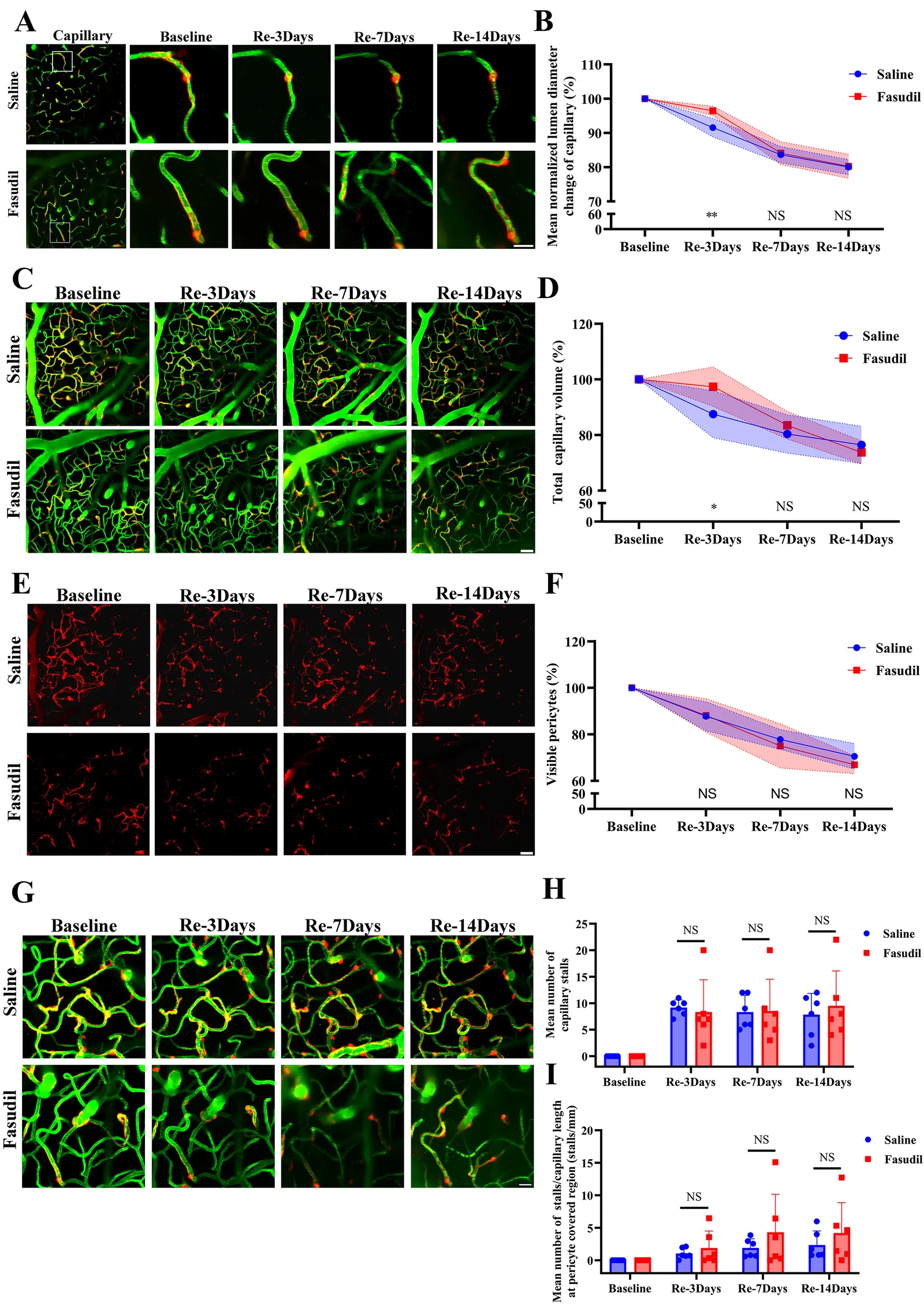
**Short-term effects of fasudil on microcirculation following tMCAO. (A)** Representative two-photon image showing changes in capillary lumen diameter following tMCAO. Scale bar = 20μm. **(B)** Quantification and comparison of mean normalized capillary lumen diameter changes at pericytes locations. **(C)** Representative image showing changes in TCV following tMCAO. Scale bar = 50μm. **(D)** Quantification and comparison of TCV changes in saline and fasudil groups. **(E)** Representative two-photon image showing changes in visible pericytes following tMCAO. Scale bar = 50μm. **(F)** Quantification and comparison of the number of visible pericytes in saline and fasudil groups. **(G)** Representative image showing changes in capillary stalls following tMCAO. Scale bar = 20 µm. **(H)** Quantification and comparison of the mean number of capillary stalls in saline and fasudil groups. (I) Quantification and comparison of the mean number of capillary stalls at pericyte covered segment in saline and fasudil groups. In panels B, D, F, H, and I, two-tailed Student’s t-tests were used for statistical. N =6 per group. * *p* < 0.05. Data are presented as mean ± SD.

Fasudil, a Rho-kinase inhibitor, has been reported to prevent pericyte constriction through its calcium-antagonistic properties in experimental stroke. However, its effect on pericyte survival following stroke has not been documented. In this study, we investigated whether administering fasudil (10 mg/kg intraperitoneally, daily) could prevent pericyte loss and improve capillary perfusion after stroke. Our findings showed that fasudil provided limited, short-term benefits in stroke models by dilating the vessel lumen (Figs. 5A, 5B) compared to the saline group on day 3 post-tMCAO. Fasudil also increased total capillary volume on day 3 (Figs. 5C, 5D). However, these effects were not observed on day 7 and 14 post-tMCAO. Furthermore, fasudil did not significantly affect the mean number of capillary stalls, pericyte-associated capillary stalls, or the number of visible pericytes at any observation time point (Figs. 5E-I). These results suggest that while fasudil elicited an early response from surviving pericytes following stroke, it did not alter their eventual fate. This lack of long-term effect may contribute to the progression of irreversible microcirculation dysfunction.

### Pericyte necroptosis contributed to delayed pericyte loss in mouse experimental stroke

**Figure 6.**
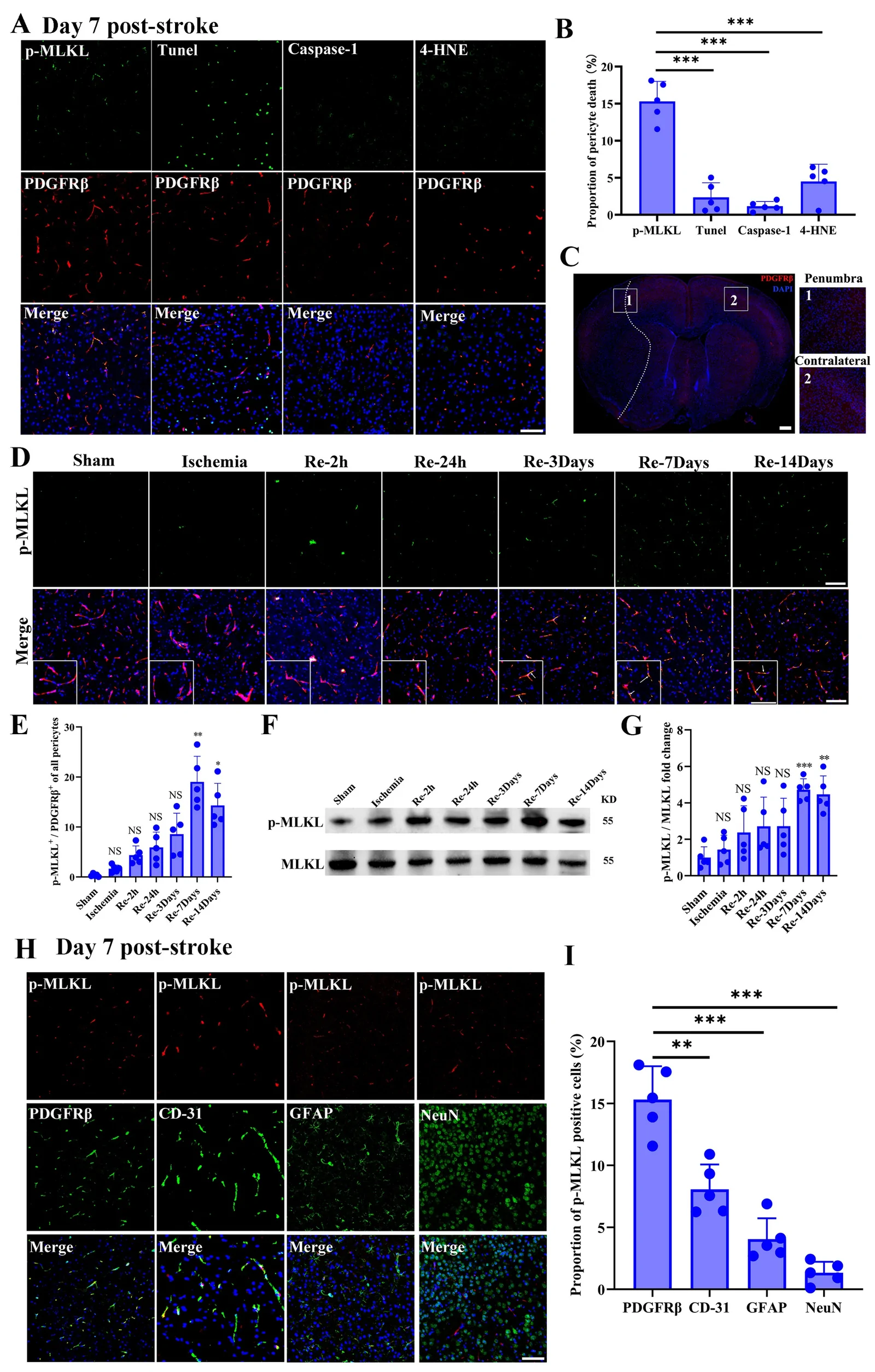
**Necroptosis predominantly occurs in pericytes following tMCAO. (A)** Representative immunofluorescence images showing double-staining of PDGFRβ with cell death markers: necroptosis (p-MLKL), apoptosis (TUNEL), pyroptosis (Caspase-1), ferroptosis (4-HNE) at day 7 post-reperfusion. Pericytes are labeled in red, and cell death markers are shown in green. Scale bar = 50μm. **(B)** Quantification of co-localization between p-MLKL, and pericytes, and between pericytes and other cell death markers on day 7 post-reperfusion. **(C)** Schematic illustration of region of interest (ROI) selection for immunohistochemical imaging. ROI1 indicates the ipsilateral ischemic penumbra, and ROI2 represents the contralateral hemisphere. Immunostaining was performed ROI1. **(D)** Experimental timeline for assessing pericyte necroptosis following tMCAO. Enlarged views of the highlighted areas show co-localization of p-MLKL^+^ and PDGFRβ^+^ at multiple time points. White arrows indicate p-MLKL^+^/PDGFRβ^+^ cells. Scale bar = 50μm. **(E)** Quantification of p-MLKL^+^ pericytes in sham, ischemia, and at 2 hours, 24 hours, 3 days, 7 days, and 14 days post-reperfusion. **(F-G)** Representative Western blot image and corresponding quantification of p-MLKL expression normalized to MLKL in cortical tissue from the ischemic penumbra. (H-I) Representative images and quantification of co-localization between p-MLKL^+^ and CD31^+^ endothelial cells, GFAP^+^ astrocytes, and NeuN^+^ neurons on day 7 post-reperfusion. In panels B, E, G and I, one-way ANOVA was used for statistical. N = 5 per group. * *p* < 0.05; ** *p* < 0.01; *** *p*< 0.001. Data are presented as mean ± SD.

To explore the mechanisms underlying pericyte loss following tMCAO in mice, we examined the types of pericyte death in PDGFRβ-tdTomato mice on day 7 post-stroke (Fig. 6A). Immunohistochemical analysis revealed that 15.31% ± 2.69% of PDGFRβ^+^ pericytes in the perifocal region were co-stained with the necroptosis marker p-MLKL. In contrast, fewer than 10% of PDGFRβ^+^ pericytes were positive for markers of other cell death types, including TUNEL (apoptosis), caspase-1 (pyroptosis), and 4-HNE (ferroptosis) (Fig. 6B). To enhance reliability, we further examined the dynamics of various pericyte death pathways at multiple time points following tMCAO. Although co-localization of other cell death markers with pericytes was observed, their incidence was consistently lower than that of necroptosis, particularly on day 7 (Supplementary Fig. 9). These findings indicate that necroptosis is the predominant form of pericyte death following stroke. Quantification of p-MLKL^+^/PDGFRβ^+^ cells over time revealed a gradual increase post-stroke, peaking at day 7 and remaining elevated through day 14 (Figs. 6D, E). Western blot analysis confirmed a significant elevation in the p-MLKL/MLKL ratio in the perifocal region at both day 7 and 14 post-tMCAO (Figs. 6F, G). Double immunofluorescence for cell-specific markers and p-MLKL revealed that the proportion of p-MLKL double-positive cells was significantly higher in PDGFRβ^+^ cells compared to CD31^+^ (endothelial cells), GFAP^+^ (astrocytes), and NeuN^+^ (neurons) (Figs. 6H, I). These results suggest that pericyte necroptosis, peaking around day 7 post-stroke, plays a major role in delayed pericyte loss in the tMCAO mouse model.

### Combined therapy with Nec-1 and fasudil improved microcirculation and neurological outcomes

**Figure 7.**
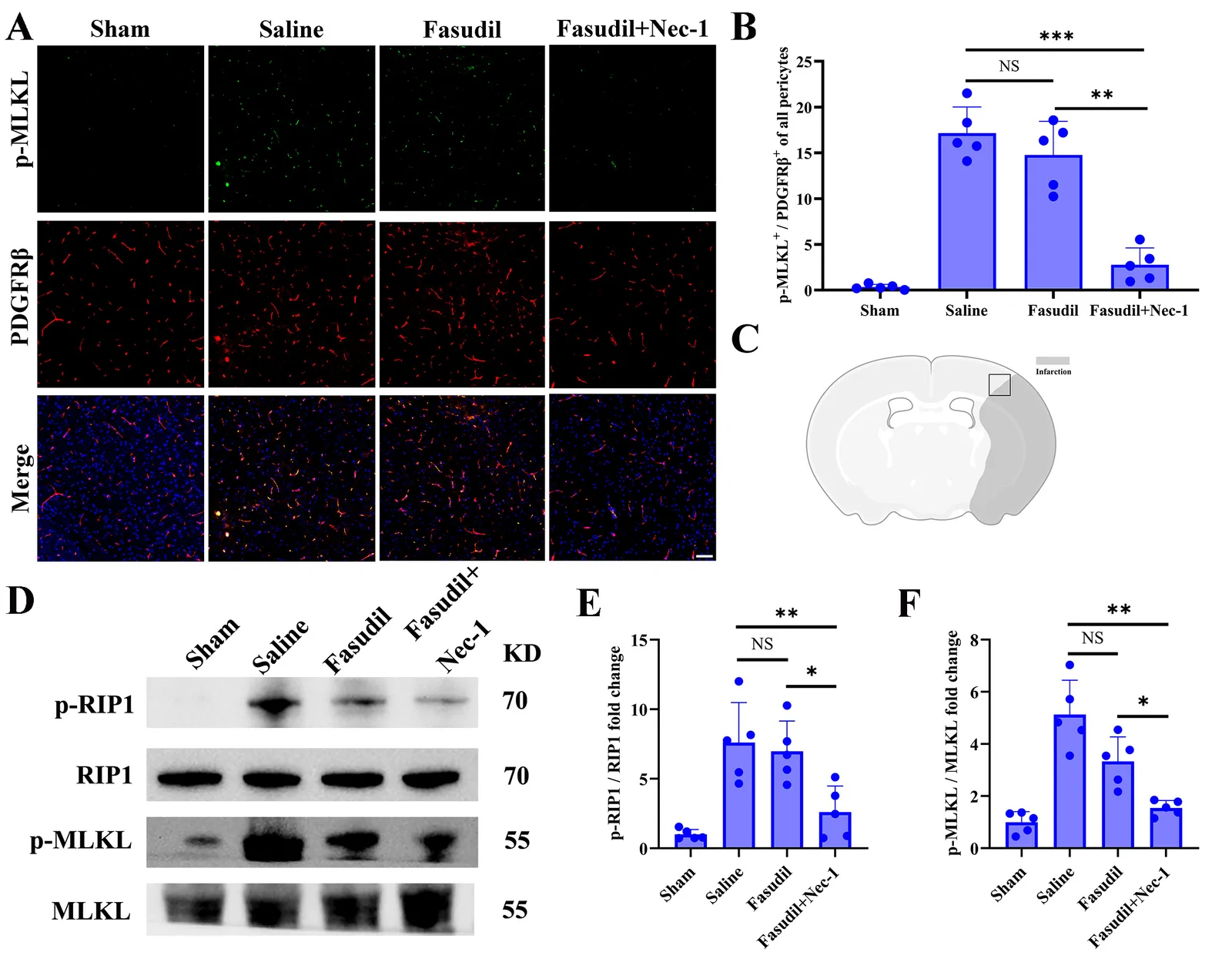
**Effects of fasudil combined with Nec-1 on reducing pericyte necroptosis following tMCAO. (A-B)** Representative immunofluorescence images and quantification of p-MLKL^+^/PDGFRβ^+^ cells on day 7 post-reperfusion. Scale bar = 50μm. **(C)** Schematic illustration of the ischemic penumbra zone. The gray area represents the infarct core, and the white area indicates normal brain tissue. **(D-F)** Representative Western blot images and quantification of p-RIP1 expression normalized to RIP1, and p-MLKL expression normalized to MLKL, in cortical tissue from the ischemic penumbra zone. In panels B, E, and F, one-way ANOVA was used for statistical. N = 5 per group. * *p* < 0.05; ** *p* < 0.01; *** *p* < 0.001. Data are presented as mean ± SD.

**Figure 8.**
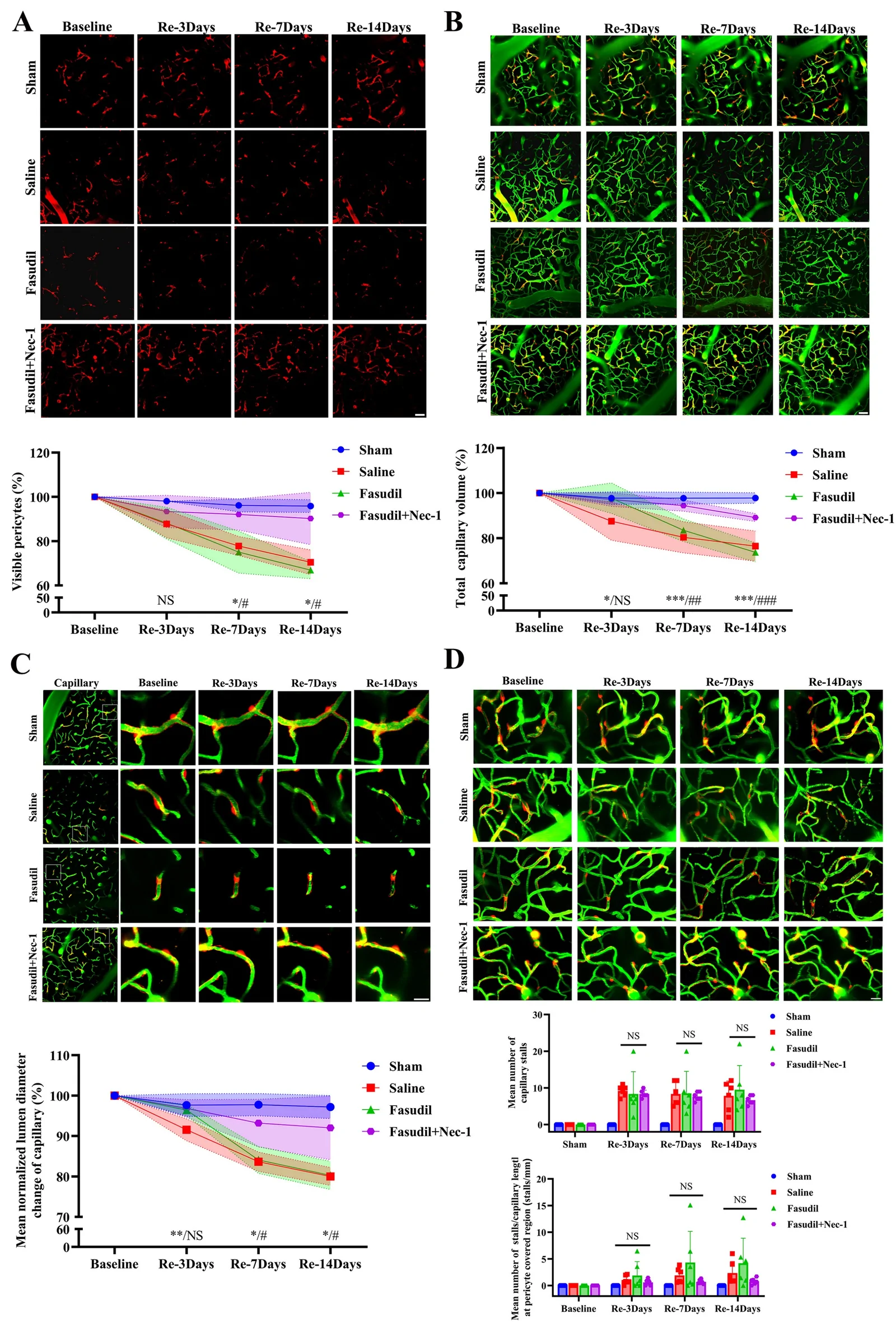
**Treatment with fasudil combined with Nec-1 improves capillary perfusion and pericyte survival during the subacute phase following tMCAO. (A)** Representative image and quantification of visible pericytes among different treatment groups. Scale bar = 50μm. **(B)** Representative image and quantification of TCV among different treatment groups. Scale bar = 50μm. **(C)** Representative image and quantification of mean normalized capillary lumen diameter among different treatment groups. Scale bar = 20μm. **(D)** Representative image and quantification of capillary stalls among different treatment groups. Scale bar = 20μm. In panels A-D, one-way ANOVA was used for statistical. N = 6 per group. * indicate comparisons between the fasudil+Nec-1 and saline groups, and # indicate comparisons between the fasudil+Nec-1 and fasudil groups. * *p* < 0.05; ** *p* < 0.01. Data are presented as mean ± SD.

Necrostatin-1 (Nec-1), a RIP1 inhibitor, blocks necroptosis by inhibiting the RIP1-mediated RIP3/MLKL pathway. In this study, we demonstrated that Nec-1 (1.65mg/kg intraperitoneally, daily) significantly reduced p-MLKL expression in capillary pericytes within the perifocal region post-stroke, compared to saline or fasudil alone (Figs. 7A–7F). Using in vivo two-photon imaging, we observed that combined therapy prevented the reduction in visible pericytes in the perifocal region, demonstrating superior improvement compared to both the saline and fasudil-alone group on day 7 and 14. In contrast, significant reductions in visible pericytes were observed in the saline and fasudil-alone groups starting from day 3 post-tMCAO (Fig. 8A). Similarly, the combined therapy preserved total capillary volume, showing significant differences compared to the saline group and fasudil-alone group on day 7 and 14 post-tMCAO. Fasudil alone maintained capillary volume only until day 3, whereas the saline group exhibited a progressive reduction over time (Fig. 8B). This trend was also reflected in capillary lumen diameter and pericyte-associated capillary stalls, but not in the mean number of capillary stalls (Figs. 8C, D). These findings suggest that Nec-1 prevents post-ischemic pericyte loss by inhibiting necroptosis, thereby allowing surviving pericytes to respond to fasudil and reperfusion, ultimately contributing to microcirculation restoration.

### Neurological and microvascular benefits of combined therapy

**Figure 9.**
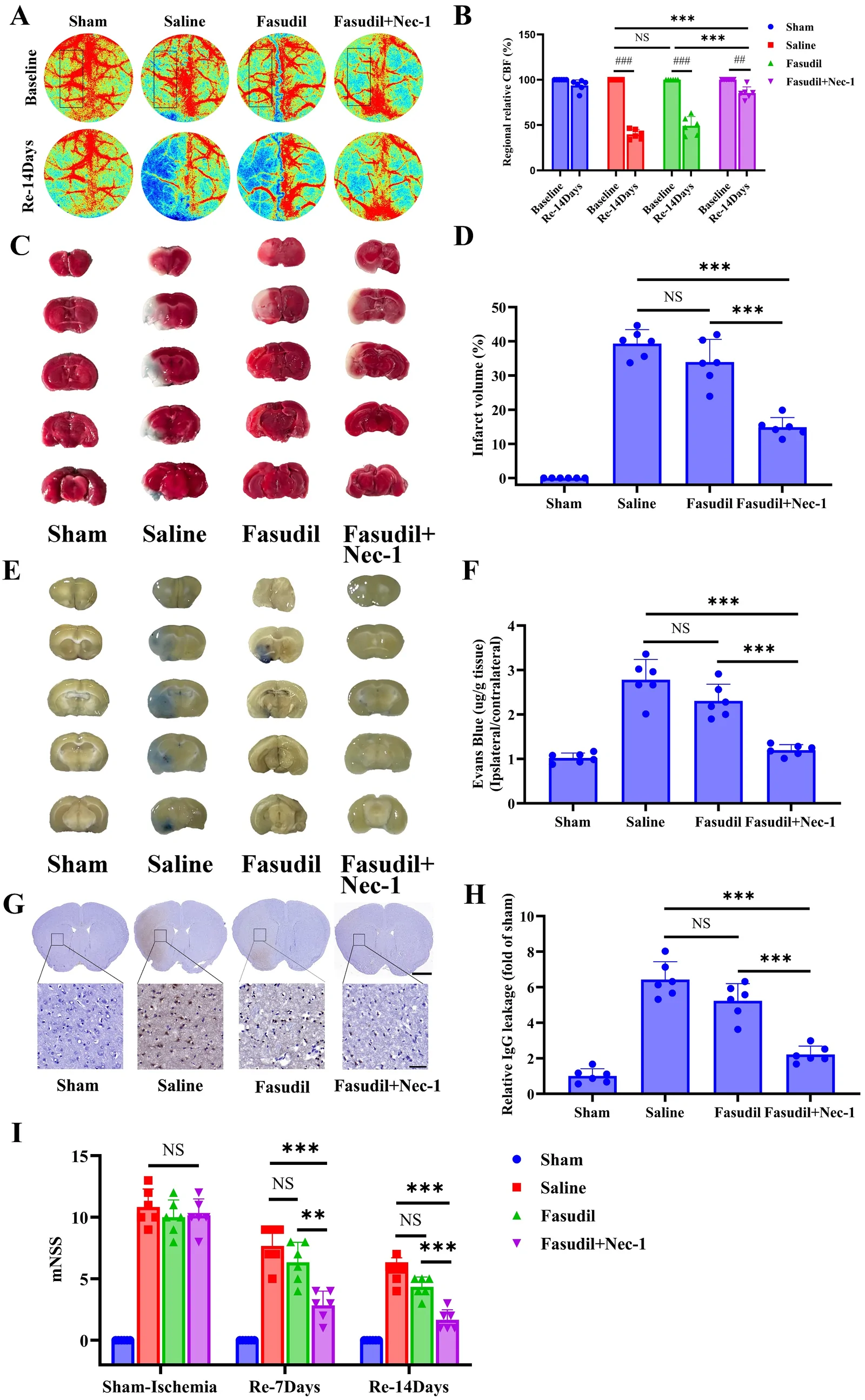
**Therapeutic effects of fasudil combined with Nec-1 on cerebral injury following tMCAO. (A)** Representative LSCI images of cerebral blood flow in different groups at baseline and at day 14 post-reperfusion. **(B)** Quantification of regional cerebral blood flow in the ischemic penumbra among different groups. **(C)** Representative TTC-stained brain slices showing infarct regions. **(D)** Quantification of cerebral infarct volume. **(E)** Representative brain slices showing Evans blue extravasation; blue areas indicate regions of dye leakage. **(F)** Quantification of Evans blue content in brain tissue. **(G)** Representative immunohistochemical images of IgG extravasation. Higher magnifications views of the boxed areas are shown below, highlighting IgG-positive regions. (H) Semi-quantitative analysis of IgG integrated optical density (IOD) to assess BBB integrity among different groups. **(I)** Modified neurological severity score (mNSS) assessed during ischemia, and on day 7 and 14 post-reperfusion. In panel B, # indicates comparisons between baseline and day 14 post-reperfusion within each group using paired-tests. In panels B, D, F, H, and I, * indicates comparisons among different treatment groups using one-way ANOVA. N = 6 per group. * *p* < 0.05, ** *p* < 0.01, *** *p* < 0.001. Data are presented as mean ± SD.

Laser speckle contrast imaging (LSCI) on day 14 revealed that combined therapy improved blood flow in the perifocal region compared to either the saline or fasudil-alone groups (Figs. 9A, 9B). Infarct volume was also significantly reduced in the combined therapy group compared to both fasudil and saline groups on day 3 (Figs. 9C, 9D). Blood-brain barrier (BBB) disruption, as measured by Evans blue and IgG extravasation, was significantly decreased in the combined therapy group (Figs. 9E-H). Neurological function, evaluated using the mNSS, showed significant improvement in the combined therapy group on both day 7 and 14 (Fig. 9I). These findings highlight the synergistic effects of Nec-1 and fasudil in preserving pericytes, improving microcirculation, and enhancing neurological outcomes after experimental stroke.

## DISCUSSION

It has been previously demonstrated that excessive constriction and subsequent loss of capillary pericytes following cerebral ischemia contribute to the "no-reflow" phenomenon in the perifocal region despite early proximal vessel recanalization [8]. The dynamics of post-ischemic pericyte dysfunction have been reported in only a few studies, and the mechanisms of pericyte loss remain unclear. In the current study, we performed repeated in vivo observations on pericyte dysfunction in mice subjected to tMCAO over 14 consecutive days. We found that pericytes in different vessel segments underwent distinct, dynamic, and reversible changes in their function and numbers during ischemia-reperfusion. Using multivariate analysis, we showed for the first time that pericyte loss on day 7 was significantly associated with poor outcomes, including incomplete restoration of microcirculation and increased mortality, after vessel recanalization in tMCAO mice. Furthermore, we revealed that pericyte necroptosis, starting on day 7 after ischemic stroke, was responsible for delayed pericyte loss, which contributed to the worsening of microcirculation in the perifocal region during the subacute phase of ischemic stroke.

The mechanism underlying the "no-reflow" phenomenon in acute ischemic stroke remains elusive despite extensive exploration since its initial proposal half a century ago [15]. Recent advances, including the use of two-photon microscopy for in vivo imaging of capillary perfusion in reperfused mouse brains, have proven invaluable in studying the no-reflow phenomenon [10, 16, 17]. These techniques allow for repeated in vivo observation of microcirculation in stroke models. In our study, we performed the longest reported in vivo observation of capillary perfusion and pericyte survival in a stroke model, spanning 14 days. We observed that the number of perfused capillaries decreased by approximately 30% immediately after MCA occlusion and that capillary perfusion remained significantly impaired 2 hours after reperfusion, recovering to baseline levels by 24 hours. A second phase of perfusion decline was observed on day 3, progressively worsening over the 14-day observation period.

The delayed, temporary recovery of capillary perfusion in the early phase after reperfusion aligns with previous reports. For instance, Qiu et al. reported substantial constriction of microcirculation vessels, including precapillary arteries, capillaries, and post-capillary venules, at 3 hours after reperfusion, with dilation to baseline levels observed by 24 hours [17]. Shrouder et al. extended these observations to 7 days, noting trends in capillary diameter and perfusion changes similar to our findings [10]. Collectively, these results suggest that the no-reflow phenomenon begins with vasodynamic instability in the microcirculation shortly after recanalization and continues to deteriorate during the acute and subacute phases of stroke, potentially impairing neurovascular unit restoration and stroke recovery.

The persistent constriction of capillary pericytes followed by pericyte death during ischemia-reperfusion has been shown to play a critical role in capillary perfusion failure despite upstream vessel recanalization [8, 9, 12]. Our study confirmed the extensive role of pericyte constriction in regulating capillary perfusion after recanalization. Notably, we found that reduced pericyte numbers on day 7 were an independent risk factor for poor capillary perfusion outcomes on day 14. This underscores the critical importance of pericyte survival in sustained microcirculation function after stroke.

While previous studies have reported pericyte loss occurring rapidly within 24 hours following stroke, our study revealed a delayed, progressive decline in the number of visible pericytes occurring on day 7. This discrepancy may result from differences in observation times, ischemia durations, or study methodologies. It is noted that a transient and significant increase in the number visible pericytes was observed soon after ischemia. Several explanations were considered. Firstly, this may have been due to increased PDGFRβ expression following pericytes activation, which is consistent with a recent report by Roth [18]. Secondly, pericyte migration may also have contributed to the increased pericyte numbers, as this has been reported to occur within 1-2 hours after stroke [19, 20]. Thirdly, tissue compression attributed by reduced perfusion may be another reason for increased visible pericytes. Our findings suggest that surviving pericytes respond rapidly to ischemic insults and may play key roles in regulating capillary circulation, early interventions targeting pericyte function could hold promise for promoting stroke recovery.

Persistent capillary pericyte constriction followed by pericytes death in rigor after cerebral ischemia-reperfusion has been observed in previous studies [8, 9]. Pericyte constriction has been found to be mediated by excessive intracellular calcium concentration [9, 13, 21]. Many investigations using calcium antagonists to prevent pericytes constriction have shown vessel dilation and improved capillary perfusion in experimental stroke [9, 10, 22]. However, most studies evaluated immediate or short-term efficacy of the intervention, whether calcium antagonists bring sustained benefit to the capillary circulation remains undetermined. Early clinical studies investigating the effects of calcium antagonists on the outcomes of stroke patients have shown inconsistent results [23-26]. A recent meta-analysis found that adding calcium antagonists to the treatment regimen did not improve clinical outcomes including mortality and dependence, in stroke patients [27]. We found that fasudil monotherapy did not improved capillary perfusion or prevent pericytes loss on day 14, although increased capillary perfusion was observed on day 3. This indicated that alternative mechanisms are involved in pericytes loss after reperfusion. The transient recovery of capillary perfusion on day 3 may be attributed to the response of surviving pericytes to fasudil. In order to explore the mechanism of pericytes loss, we investigated the form of pericyte death on day 7 after reperfusion, and found that necroptosis instead of apoptosis, pyroptosis, or ferroptosis, accounted for the majority of pericyte death in the subacute phase of stroke.

Necroptosis, a regulated form of necrotic cell death mediated by receptor-interacting protein kinases (RIPK) 1, RIPK3, and mixed lineage kinase domain-like pseudokinase (MLKL) [28], was identified as the predominant mechanism of pericyte death in our study. Necroptosis has been shown to contribute to neural cell death in the penumbra region after stroke, particularly when apoptosis is suppressed [28, 29]. In experimental stroke, necroptosis-related signaling proteins are upregulated in neurons. Pharmacological or genetic inhibition of RIPK1 significantly reduced ischemic brain injury [30-32]. Pericytes necroptosis has been investigated in only a few studies. Research on experimental intracerebral hemorrhage showed that RIPK1, but not MLKL was activated in pericytes, and RIPK1 inhibition reduced brain edema and BBB permeability [33]. A recent study found that pericytes, neurons and astrocytes subjected to oxygen-glucose deprivation shared similar genes expression pathways involving necroptosis [34]. These results, together with our findings, suggested that targeting pericyte necroptosis may be a promising neuroprotective strategy in ischemic stroke. The therapeutic effects and mechanisms of Nec-1 in experimental stroke have been demonstrated in previous studies [31, 32]. For the first time, we showed the combined therapy with fasudil and Nec-1 significantly prevented pericyte loss and improved capillary perfusion on day 14, compared with either fasudil monotherapy or saline. Fasudil's calcium-antagonistic properties likely contributed to short-term perfusion improvements, increasing Nec-1 accessibility to hypo-perfused regions, while Nec-1 inhibited necroptosis, preserving pericytes throughout the acute and subacute phases of stroke. This synergistic approach enhanced neurovascular unit restoration and neurological outcomes. It should be noted that fasudil and Nec-1 were administered intraperitoneally rather than via stereotaxic injection. This method of delivery suggests potential for clinical translatability via intravenous administration, which is less invasive and allows for repeated dosing. However, concerns have been raised that systemic administration of fasudil may lead to vascular steal in the contralateral hemisphere. Although early investigation showed that nimodipine improved perfusion in the ischemic brain without causing vascular steal [35], a recent study reported that infusion of hydrazine, a vasodilating agent, improved CBF in poorly collateralized stroke models but not in well-collateralized ones, implying possible vascular steal [36]. In our study, worsening perfusion in the penumbra region was not observed in the fasudil group compared to the saline group at any observation time point. Future studies should further evaluate effects of fasudil on perfusion in specific brain regions.

Our study provides new insights into the mechanisms underlying the no-reflow phenomenon and highlights a promising therapeutic strategy targeting excessive constriction and necroptosis of capillary pericytes. The study's strengths include a prolonged 14-day in vivo observation period, the use of a synergistic combination therapy, and the administration of Nec-1 via a clinically translatable route. Nevertheless, our study has limitations. We cannot exclude the possibility that the observed protective effects of the combined therapy may also involve other cell types in the penumbra, such as neurons and endothelial cells. Future research using brain pericyte-deficient mouse models is warranted to confirm the specific contribution of pericyte protection to the observed outcomes.

In summary, pericyte-mediated capillary perfusion dysfunction after recanalization progresses throughout the acute and subacute phases of stroke. Pericyte necroptosis is a key contributor to irreversible microcirculation failure in the subacute phase. Combined therapy targeting excessive constriction and necroptosis of capillary pericytes offers a novel approach to improving microcirculation and neurological outcomes in ischemic stroke.

## Supplementary Materials

The Supplementary data can be found online at: www.aginganddisease.org/EN/10.14336/AD.2025.0197.
