# Supplementary material for: Microcirculation Dysfunction in Subacute Stroke: The Role of Delayed Capillary Pericyte Loss
Source: Aging Dis. 2025 Jun 4;17(3):1516–33. doi: 10.14336/AD.2025.0197 (PMC13061558; doi:10.14336/AD.2025.0197)
Supplement: Supplementary file 1 — The Supplementary data can be found online at: www.aginganddisease.org/EN/10.14336/AD.2025.0197. [file AD-17-3-1516-s.pdf]

SUPPLEMENTARY DATA

Microcirculation Dysfunction in Subacute Stroke: The Role of Delayed Capillary Pericyte Loss

Yiya Xu, Chao Chen, Jilin Weng, Ting Chen, Yingchao He, Zhiwei Song, Yinzhou Wang

© 2025. Xu Y et al. Published online at http://www.aginganddisease.org/EN/10.14336/AD.2025.0197

SUPPLEMENTARY DATA

Supplementary Table 1.

Antibodies	Source	Identifier
Anti-phospho-MLKL (Ser345) antibody	Sigma-Aldrich	MABC1158
Anti-phospho-RIP1 (Ser166) antibody	ThermoFisher	PA5-104645
Anti- MLKL antibody	Proteintech	66675-1-1g
Anti- RIP1 antibody	Immunoway	YN1850
Anti- Caspas-1 antibody	Abcam	ab138483
Anti-4 Hydroxynonenal antibody	Abcam	ab48506
Anti-CD31 antibody	R&D	AF3628-SP
Anti-GFAP antibody	Abcam	ab302644
Anti-Neun antibody	Cell Signaling Technology	24307
Tunel(In situ Cell Death Detection Kit, Fluorescein)	Roche	11684795910
Second antibodies alexa fluor 594-conjugated goat anti-rabbit IgG	Abcam	ab150080
Second antibodies alexa fluor 488-conjugated goat anti-rabbit IgG	Abcam	ab150070
Second antibodies alexa fluor 647-conjugated goat anti-mouse IgG	Abcam	ab150115
Second antibodies alexa fluor 488-conjugated goat anti-mouse IgG	Abcam	ab150113
Second antibodies alexa fluor 594-conjugated donkey anti-goat IgG	Abcam	ab150129

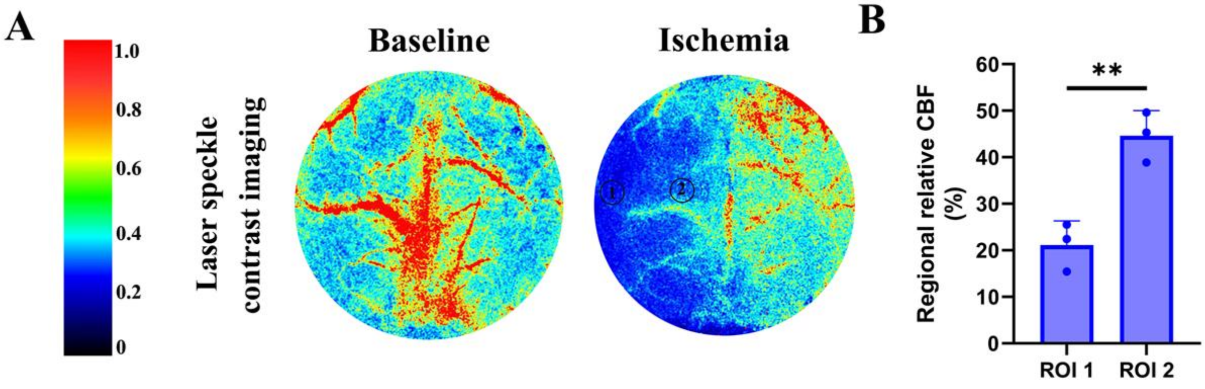

Supplementary Figure 1. Localization of the ischemic penumbra for cranial window positioning using laser speckle contrast imaging. (A) Representative laser speckle contrast image of the tMCAO mouse model. ROI 1 represents the ischemic core (CBF < 30% of baseline), while ROI 2 represents the ischemic penumbra (CBF between 30% and 50% of baseline). (B) Quantification of regional relative CBF in the ischemic core and penumbra as shown in (A). Two-tailed Student's t-tests were used for statistical analysis. N=3 per group. ** $P < 0.01$. Data are presented as mean ± SD.

© 2025. Xu Y et al. Published online at http://www.aginganddisease.org/EN/10.14336/AD.2025.0197

SUPPLEMENTARY DATA

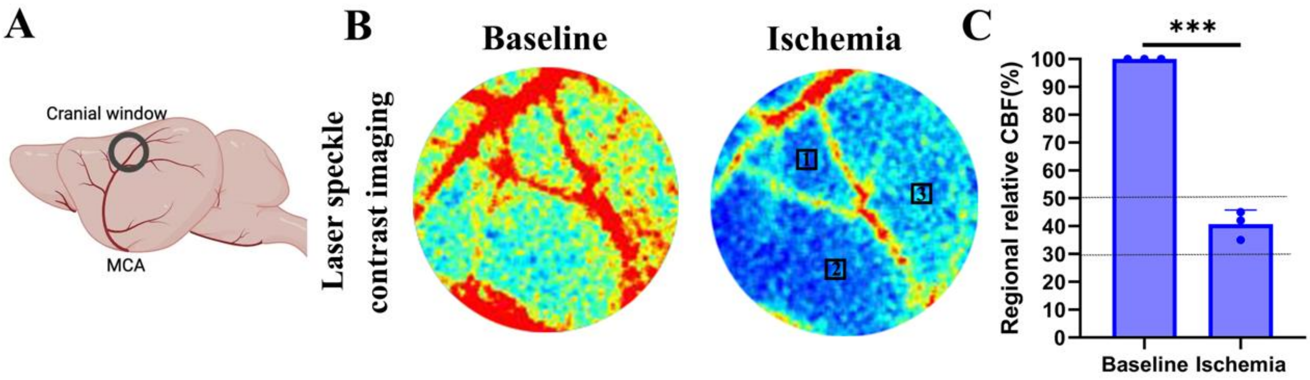

Supplementary Figure 2. Assessment of regional relative CBF by LSCI through chronical cranial window. (A) Visualization of the location of the cranial window (black circle) and the cortical branch of MCA. (B) Left: Representative image of regional relative CBF of baseline. Right: Representative image of regional relative CBF during ischemia. The square frame representative the ROI selected for two-photon imaging. (C) Quantification of regional relative CBF at baseline and during ischemia through the cranial window. Two-tailed Student's t-tests were used for statistical analysis. N=3 per group. *** *P* < 0.001. Data are presented as mean ± SD.

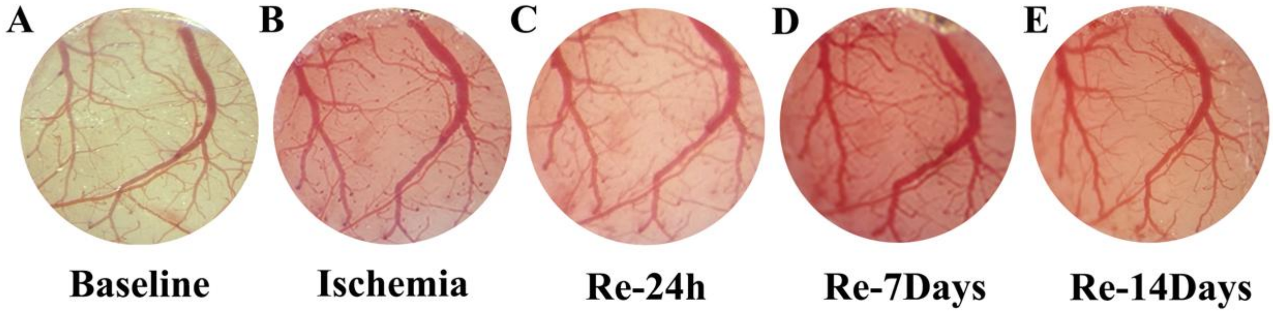

Supplementary Figure 3. Cranial window view with visible MCA branches. (A-E) Representative images of the cranial window at baseline, ischemia, Re-24h, Re -7Days and Re -14Days.

© 2025. Xu Y et al. Published online at http://www.aginganddisease.org/EN/10.14336/AD.2025.0197

SUPPLEMENTARY DATA

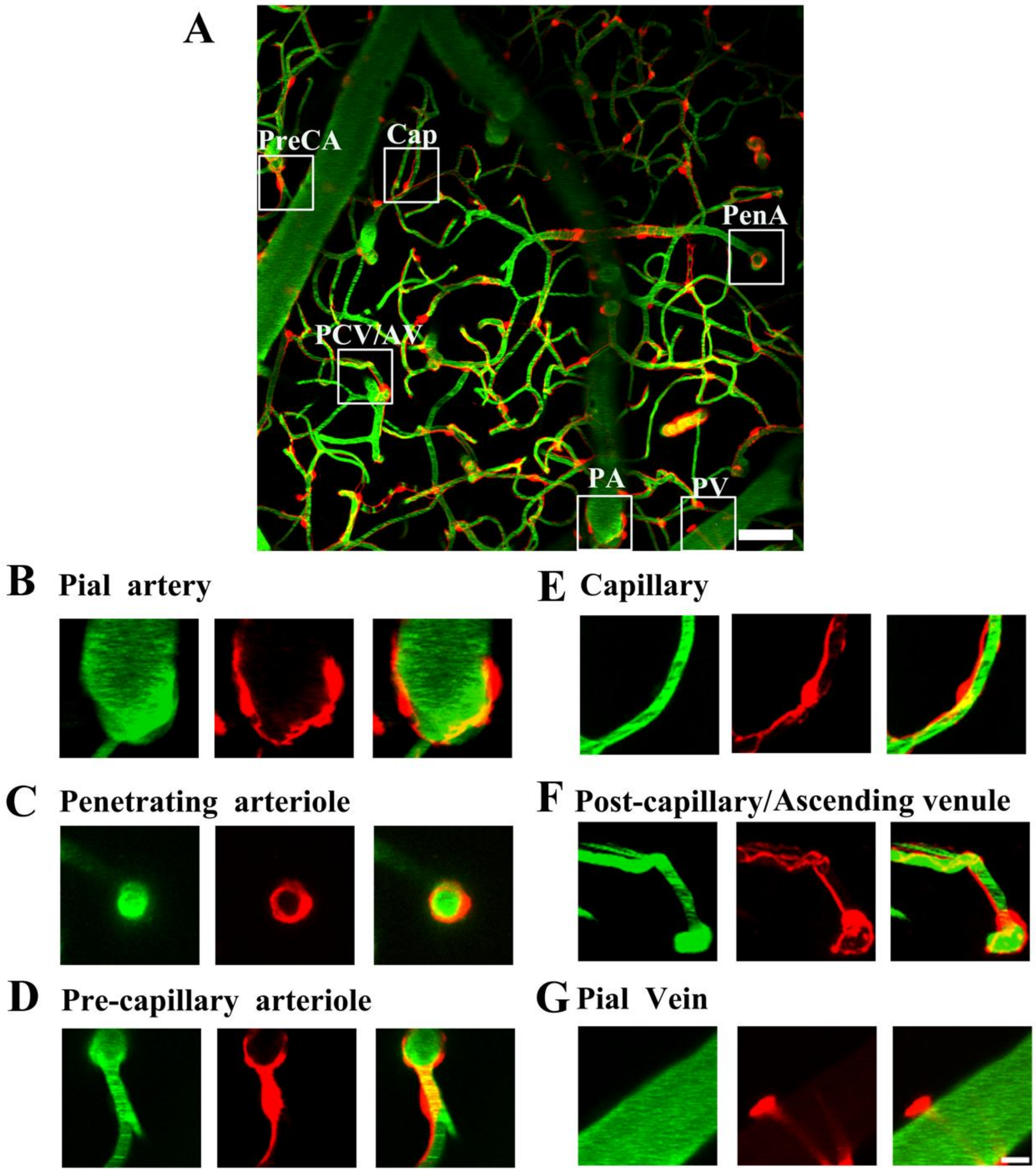

Supplementary Figure 4. In vivo two-photon imaging of the cerebral vascular hierarchy in mice. (A) Z-stack imaging of the cerebrovascular network and mural cells. Scale bar = 50µm. (B-G) Representative two-photon images of vascular segments, including the pial artery, penetrating arteriole, pre-capillary arteriole, capillary, post-capillary venule, ascending venule, and pial vein. Scale bar = 10µm. Abbreviations: PA: Pial artery; PenA: Penetrating arteriole; PreCA: Pre-capillary arteriole; Cap: Capillary; PCV: Post-capillary venule; AV: Ascending venule; PV: Pial vein

© 2025. Xu Y et al. Published online at http://www.aginganddisease.org/EN/10.14336/AD.2025.0197

SUPPLEMENTARY DATA

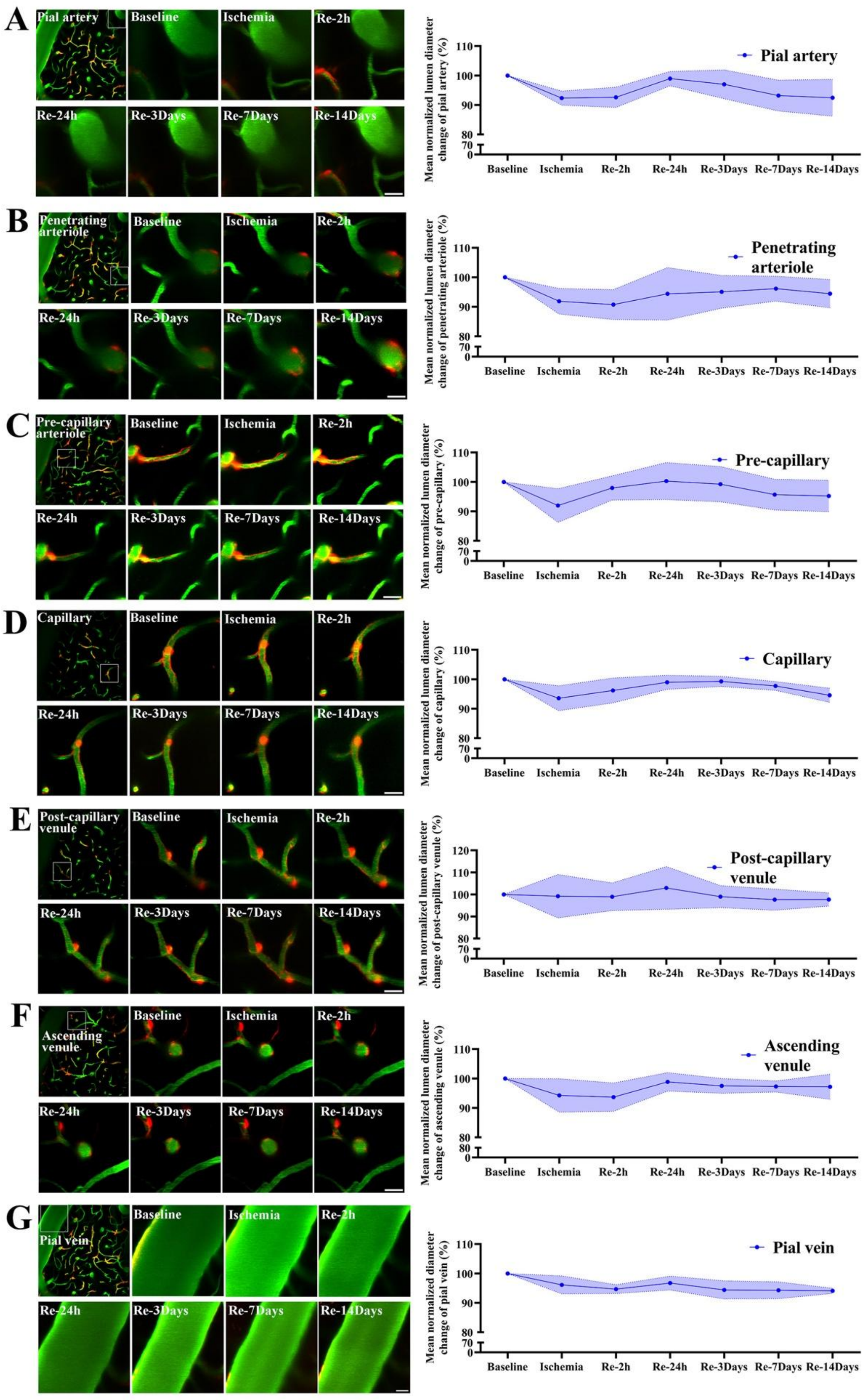

© 2025. Xu Y et al. Published online at http://www.aginganddisease.org/EN/10.14336/AD.2025.0197

SUPPLEMENTARY DATA

Supplementary Figure 5. Assessment of vascular segment diameter changes in sham mice using two-photon imaging. (A-G) Left panels: Representative two-photon images showing changes in different vascular segments, including the pial artery, penetrating arteriole, pre-capillary arteriole, capillary, post-capillary venule, ascending venule, and pial vein within the region of interest at seven time points. Scale bar = 20μm. Right panels: Quantification and comparison of mean normalized lumen diameter changes across different vascular segment. * A linear mixed-effects model was used to compare each post-stroke time point to baseline in the stroke group. N = 4 for the sham group. Data are presented as mean ± SD.

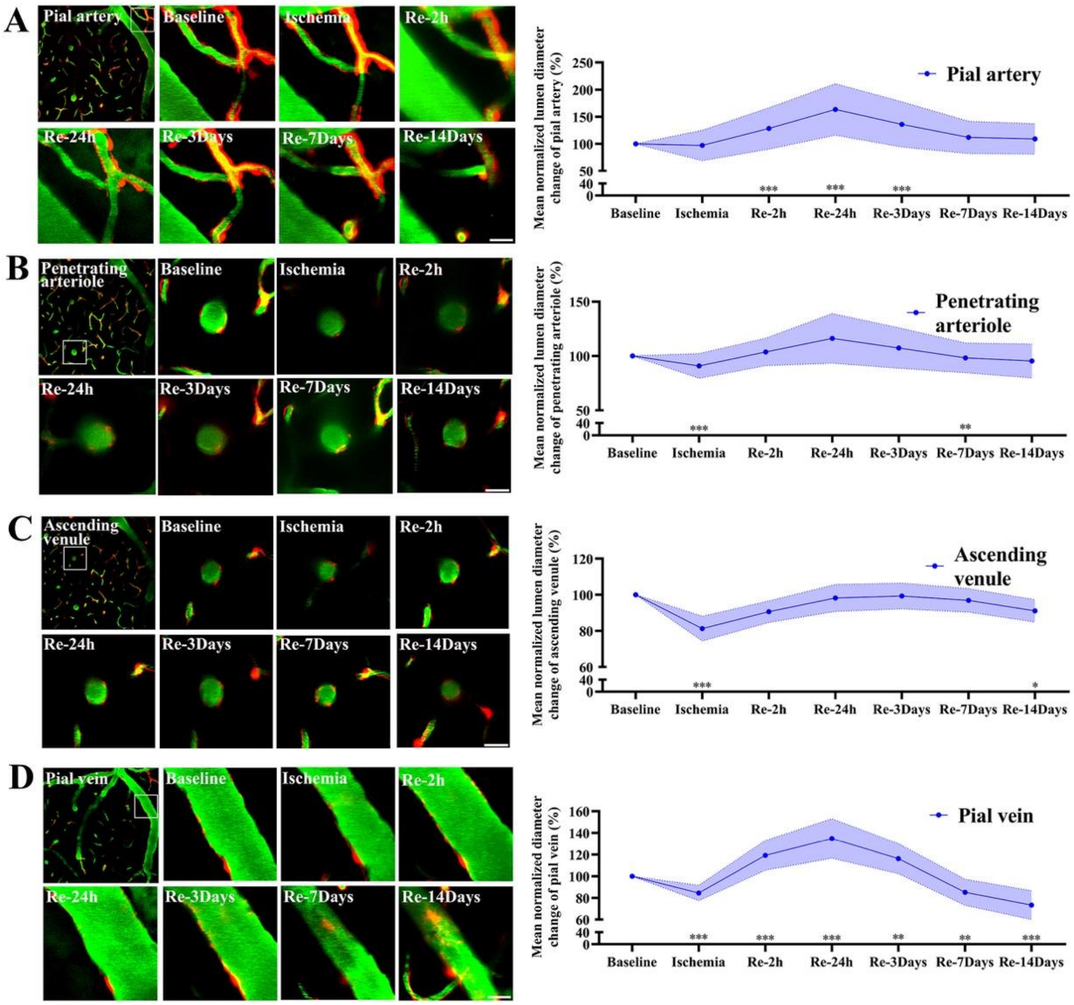

Supplementary Figure 6. Assessment of vascular segment diameter changes in stroke mice using two-photon imaging. (A-D) Left panels: Representative two-photon images showing changes in different vascular segments, including the pial artery, penetrating arteriole, ascending venule, and pial vein within the region of interest at seven time points. Scale bar = 20μm. Right panels: Quantification and comparison of mean normalized lumen diameter changes across different vascular segment. * A linear mixed-effects model was used to compare each post-stroke time point to baseline in the stroke group. N = 16 for the sham group. Data are presented as mean ± SD.

© 2025. Xu Y et al. Published online at http://www.aginganddisease.org/EN/10.14336/AD.2025.0197

SUPPLEMENTARY DATA

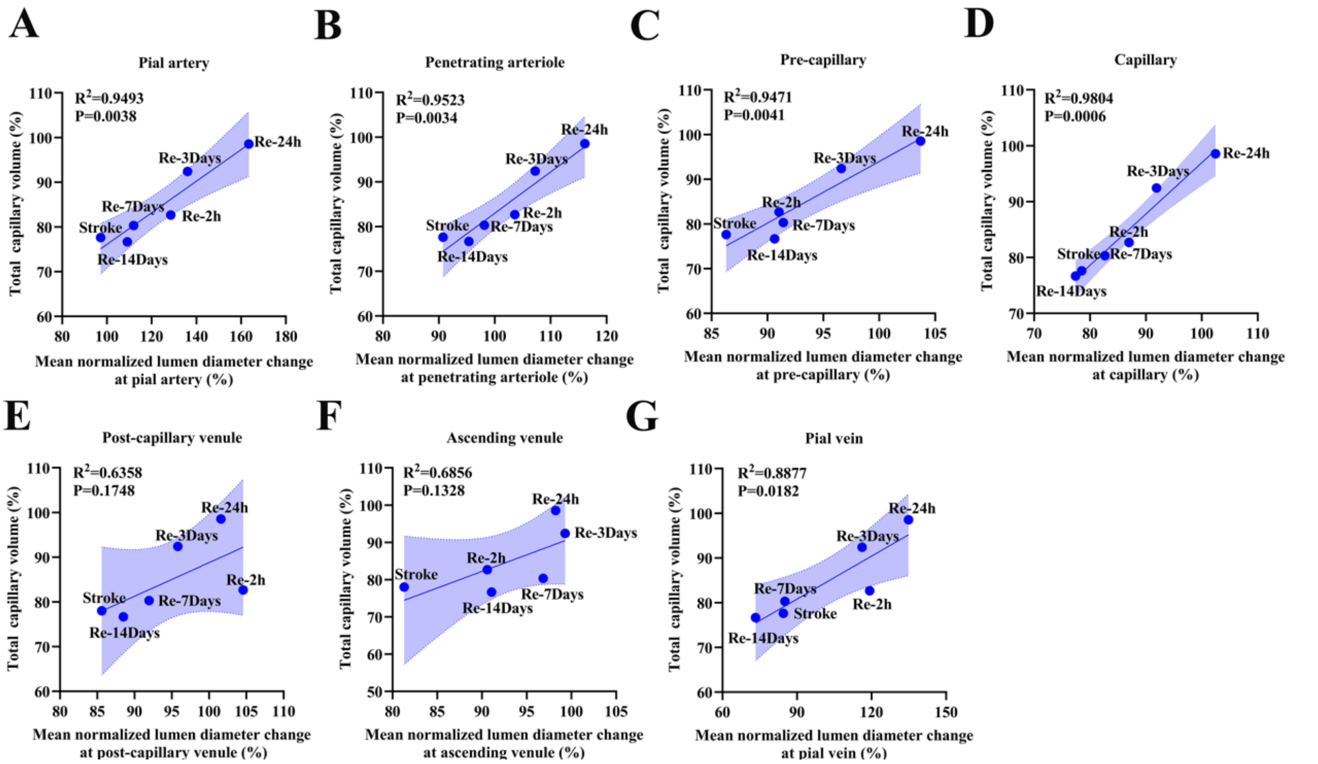

Supplementary Figure 7. Correlation between lumen diameter changes in different vascular segments and total capillary volume following tMCAO. (A-G) Pearson correlation analysis between mean lumen diameter changes of the pial artery, penetrating artery, pre-capillary arteriole, capillary, post-capillary venule, ascending venule, and pial vein and total capillary volume (TCV). The correlation coefficient (R) and *p*-value are indicated for each segment. N = 16 for the stroke group. Data are presented as mean ± SD.

SUPPLEMENTARY DATA

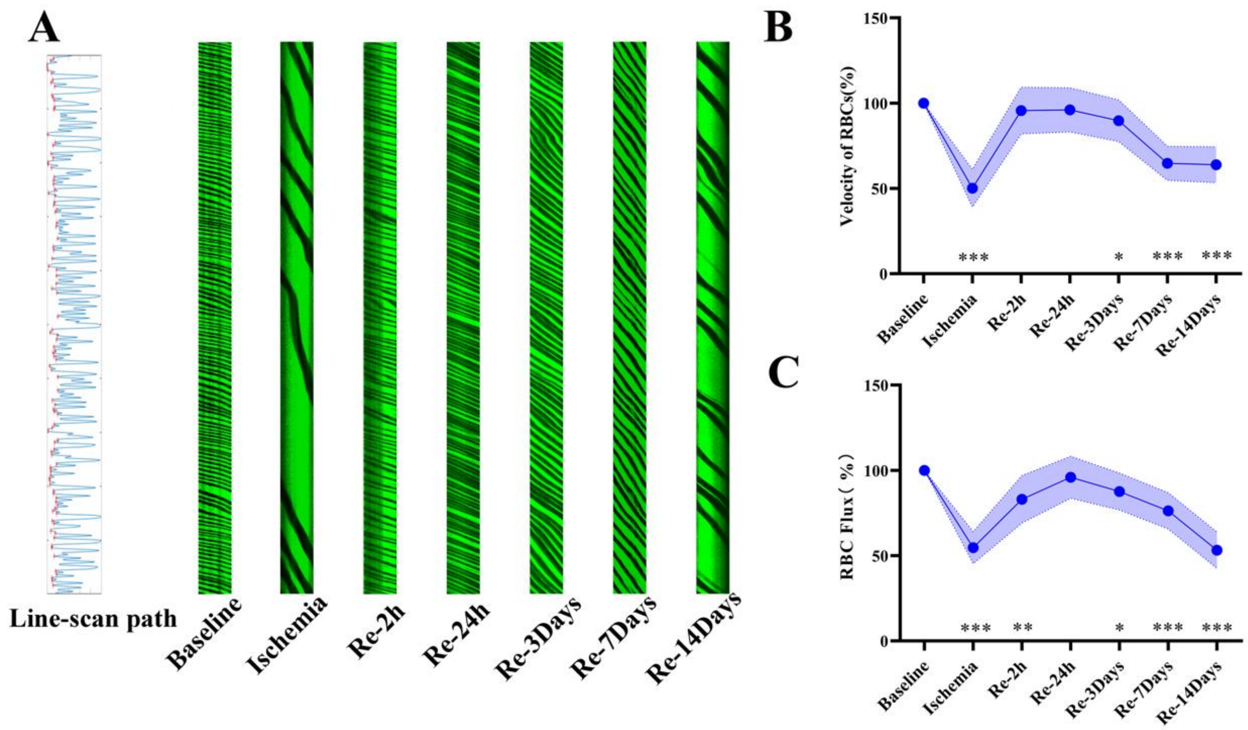

Supplementary Figure 8. Pericyte-mediated changes in capillary blood flow velocity and flux after tMCAO assessed by two-photon imaging. (A) Left panel: Representative RBC scan paths along capillaries at pericyte locations. △t： Represents the time interval in milliseconds(ms). △x： Represents the spatial displacement of blood cells. Blood cell velocity = △x/△t. Blood cell flux = cells per second. Right panel: Representative blood flow data obtained from line-scan imaging. For each mouse, blood flow was measured in 10 pericyte-associated capillaries, and the average value was used for analysis. (B) Changes in red blood cell (RBC) velocity at baseline, during ischemia, and at different reperfusion time points. (C) Changes in RBC Flux at baseline, during ischemia, and at different reperfusion time points. *A linear mixed-effects model was used to compare each post-stroke time point to baseline within the stroke group. N=16 for the stroke group. *$P < 0.05$; **$P < 0.01$; ***$P< 0.001$. Data are presented as mean ± SD.

© 2025. Xu Y et al. Published online at http://www.aginganddisease.org/EN/10.14336/AD.2025.0197

SUPPLEMENTARY DATA

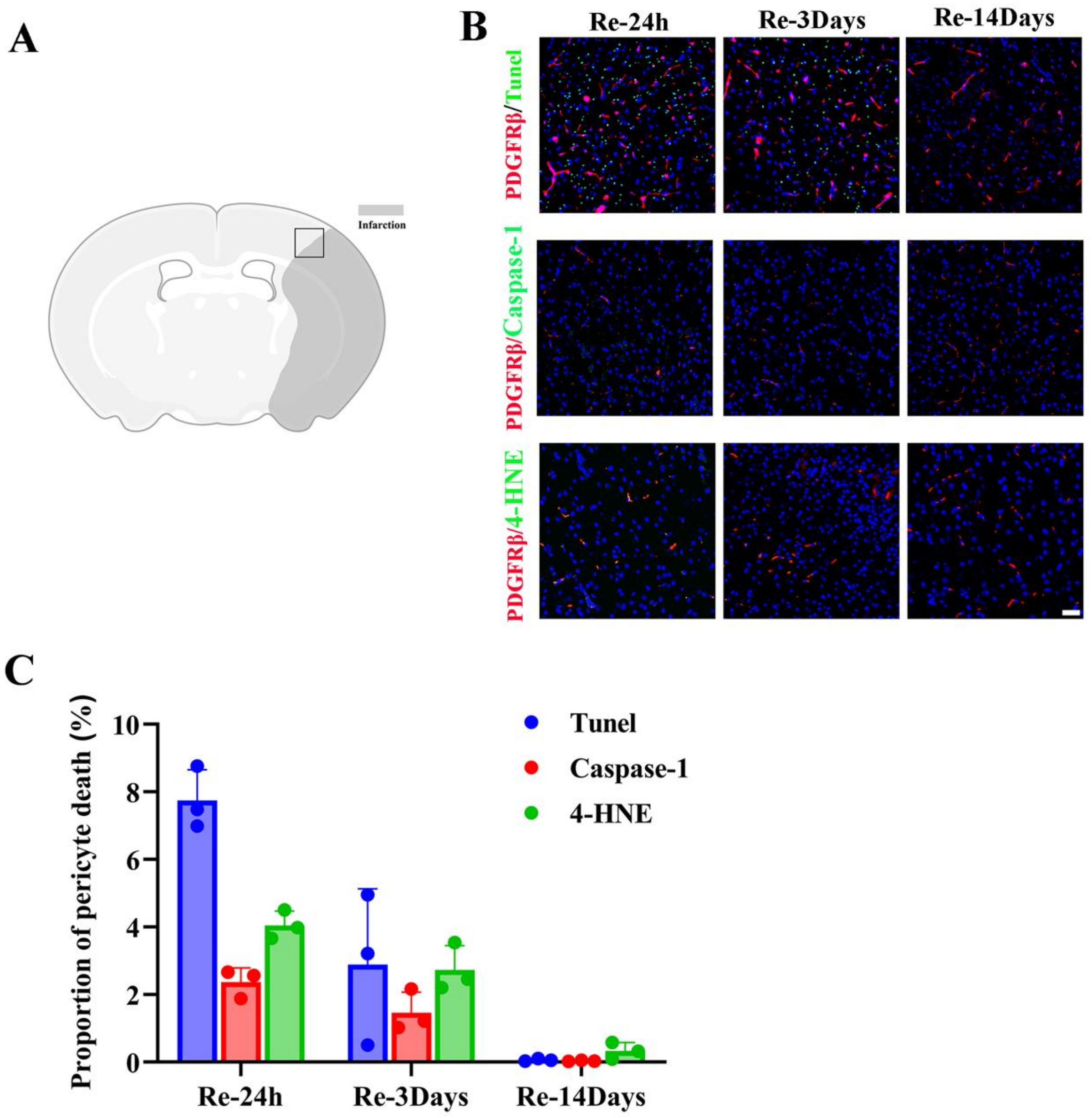

Supplementary Figure 9. Distinct forms of pericyte death at different time points following tMCAO. (A) Schematic illustration of the ischemic penumbra zone. (B) Representative immunofluorescence images showing various forms of pericyte death at 24 hours, day 3, and day 14 post-reperfusion, including TUNEL (apoptosis), Caspase-1 (pyroptosis), and 4-HNE (ferroptosis). Pericytes are labeled in red, and cell death markers are shown in green. Scale bar = 50μm. (C) Quantification of co-localization between pericytes and cell death markers at 24 hours, day 7 and day 14 post-reperfusion.

© 2025. Xu Y et al. Published online at http://www.aginganddisease.org/EN/10.14336/AD.2025.0197
